# Global airborne microbial communities controlled by surrounding landscapes and wind conditions

**DOI:** 10.1038/s41598-019-51073-4

**Published:** 2019-10-08

**Authors:** Romie Tignat-Perrier, Aurélien Dommergue, Alban Thollot, Christoph Keuschnig, Olivier Magand, Timothy M. Vogel, Catherine Larose

**Affiliations:** 1 Institut des Géosciences de l’Environnement, Univ Grenoble Alpes, CNRS, IRD, Grenoble INP, Grenoble, France; 2 Environmental Microbial Genomics, Laboratoire Ampère, Ecole Centrale de Lyon, Université de Lyon, Ecully, France

**Keywords:** Microbial ecology, Biogeography, Environmental sciences

## Abstract

The atmosphere is an important route for transporting and disseminating microorganisms over short and long distances. Understanding how microorganisms are distributed in the atmosphere is critical due to their role in public health, meteorology and atmospheric chemistry. In order to determine the dominant processes that structure airborne microbial communities, we investigated the diversity and abundance of both bacteria and fungi from the PM10 particle size (particulate matter of 10 micrometers or less in diameter) as well as particulate matter chemistry and local meteorological characteristics over time at nine different meteorological stations around the world. The bacterial genera *Bacillus* and *Sphingomonas* as well as the fungal species *Pseudotaeniolina globaosa* and *Cladophialophora proteae* were the most abundant taxa of the dataset, although their relative abundances varied greatly based on sampling site. Bacterial and fungal concentration was the highest at the high-altitude and semi-arid plateau of Namco (China; 3.56 × 10^6^ ± 3.01 × 10^6^ cells/m^3^) and at the high-altitude and vegetated mountain peak Storm-Peak (Colorado, USA; 8.78 × 10^4^ ± 6.49 × 10^4^ cells/m^3^), respectively. Surrounding ecosystems, especially within a 50 km perimeter of our sampling stations, were the main contributors to the composition of airborne microbial communities. Temporal stability in the composition of airborne microbial communities was mainly explained by the diversity and evenness of the surrounding landscapes and the wind direction variability over time. Airborne microbial communities appear to be the result of large inputs from nearby sources with possible low and diluted inputs from distant sources.

## Introduction

Microbial transport in the atmosphere is critical for understanding the role microorganisms play in meteorology, atmospheric chemistry and public health. Recently, studies have shown that up to 10^6^ microbial cells can be found in one cubic meter of air^1^ and that they might be metabolically active^2,3^. Different processes, including aerosolisation and transport, might be important in selecting which microorganisms exist in the atmosphere. For example, specific bacterial taxa (*e.g*., *Actinobacteria* and some *Gammaproteobacteria*) have been proposed to be preferentially aerosolized from oceans^4^. Once aerosolized, microbial cells enter the planetary boundary layer, defined as the air layer near the ground, directly influenced by the planetary surface, from which they might eventually be transported upwards by air currents into the free troposphere (air layer above the planetary boundary layer) or even higher into the stratosphere^5–8^. Microorganisms might undergo a selection process during their way up into the troposphere and the stratosphere^9^. Studies from a limited number of sites have investigated possible processes implicated in the observed microbial community distribution in the atmosphere, such as meteorology^1,2,10–12^, seasons^11,13–16^, surface conditions^12–14,16^ and global air circulation^11,17–20^. Most research has described the airborne microbial communities at one specific site per study. Airborne fungal communities (not only fungal spores) have frequently been overlooked, despite constituting a significant health concern for crops^21,22^ and in allergic diseases^23^. A few have initiated the investigation of microbial geographic distribution by examining regional and even continental patterns^10,16,19^, although in most cases at a few time points only. Some long-range transport has been reported between regions separated by thousands of kilometers^5,6^. Several factors, such as the local landscapes, local meteorological conditions, and inputs from long-range transport have all been cited as partially responsible for the composition of airborne microbial communities^11,14,17,24–26^, but their relative contribution remains unclear. Probabilistically, proximity should have an effect, and therefore, local Earth sources of microorganisms should contribute significantly to atmospheric microbial communities especially in the planetary boundary layer. In addition, meteorological conditions (*e.g*., wind speed and direction) might lead to different temporal variability of airborne microbial communities by mediating the relative inputs of microbial populations from the different surrounding landscapes. Our goal was to evaluate the relative importance of environmental processes on the geographical and temporal variations in airborne microbial communities. This was carried out by following changes in community structure and abundance of both airborne bacteria and fungi as well as particulate matter chemistry and local meteorological characteristics over time at nine sites around the world.

## Material and Methods

**Figure 1:**
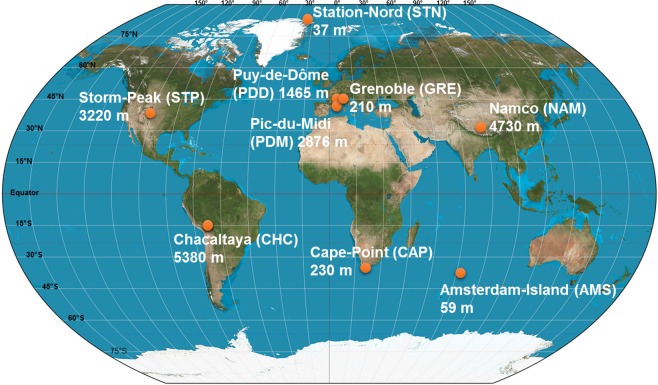
Map showing the geographical location and elevation from sea level of the nine sampling sites.

Air samples (seven to sixteen per site) were collected in 2016 and 2017 at nine sites from different latitudes (from the Arctic to the sub-Antarctica) and elevations from sea level (from 59 m to 5230 m; Fig. 1 and Supplementary Table S1). We collected particulate matter smaller than 10 µm (PM10) on pre-treated quartz fiber filters using high volume air samplers (TISCH, DIGITEL, home-made) equipped with a PM10 size-selective inlet. Quartz fiber filters were heated to 500 °C for 8 hours to remove traces of organic carbon including DNA. All the material including the filter holders, aluminium foils and plastic bags in which the filters were transported were UV-sterilized as detailed in *Dommergue et al., (2019)*^27^. A series of field and transportation blank filters were done to monitor and check the quality of the sampling protocol as presented in *Dommergue et al., (2019)*^27^.

The collection time per sample lasted one week. Depending on the site, the collected volumes ranged from 2000 m^3^ to 10000 m^3^ after standardization using SATP standards (Standard Ambient Pressure and Temperature) (Supplementary Table S1). The environment type varied from marine (Amsterdam-Island) to coastal (Cape-Point), polar (Station-Nord) and terrestrial (Grenoble, Chacaltaya, Puy-de-Dôme, Pic-du-Midi, Storm-Peak and Namco) (Table 1). For mountain peaks, we sampled at night to minimize sampling in the planetary boundary layer (the filter was left in the sampler during day time). Detailed sampling protocols are presented in *Dommergue et al., (2019)*^27^. Elemental carbon (EC), organic carbon (OC), sugar anhydrides and alcohols (levoglucosan, mannosan, galactosan, inositol, glycerol, erythriol, xylitol, arabitol, sorbitol, mannitol, trehalose, rhamnose, glucose), major soluble anions (methylsulfonic acid [MSA], SO_4_^2−^, NO_3_^−^, Cl^−^, oxalate) and cations (Na^+^, NH_4_^+^, K^+^, Mg^2+^, Ca^2+^) were analyzed^27^.

**Table 1:** Summary of bacterial and fungal abundances and bacterial (genus level) and fungal (species level) Chao1 richness estimations averaged per site and associated to a standard deviation.

Site	Name	Environment type	Coordinates and elevation from sea level	Collection start and end	Number of samples	16S rRNA gene copies/m	18S rRNA gene copies/m	Bacterial Chao1 richness estimation	Fungal Chao1 richness estimation
AMS	Amsterdam-Island, France	Marine, remote	37°47′82 ″S 77°33′04″E 59 m asl	07/09/2016 – 10/11/2016	9	1.49 × 10^5^ ± 9.17 × 10^4,a^	7.51 × 10^3^ ± 6.96 × 10^3,a’d’e’^	7.15 × 10^2^ ± 1.33 × 10^2 a^	2.25 × 10^2^ ± 4.52 × 10^1 a^’
CAP	Cape-Point Station, South Africa	Coastal	34°21′26″S 18°29′51″E 230 m asl	11/10/2016 – 05/12/2016	7	1.89 × 10^5^ ± 1.39 × 10^5 a^	1.74 × 10^3^ ± 1.21 × 10^3 a’e’^	7.64 × 10^2^ ± 4.39 × 10^2 a^	4.40 × 10^2^ ± 1.52 × 10^2 b’ d’^
STN	Station-Nord, Greenland	Polar	81°34′24″N 16°38′24″E 37 m asl	20/03/2017 – 29/06/2017	13	7.34 × 10^2^ ± 9.22 × 10^2 b^	5.24 × 10° ± 1.11 × 10^1 b’^	2.17 × 10^2^ ± 7.66 × 10^1 b^	1.04 × 10^2^ ± 5.15 × 10^1 d’^
GRE	Grenoble, France	Terrestrial, urban	45°11′38″N 05°45′44″E 210 m asl	30/06/2017 – 14/09/2017	10	1.20 × 10^6^ ± 9.38 × 10^5 ac^	5.28 × 10^4^ ± 3.61 × 10^4 d’^	7.54 × 10^2^ ± 1.05 × 10^2 a^	7.44 × 10^2^ ± 1.06 × 10^2 c’^
PDD	PuydeDôme, France	Terrestrial, continental, mountain peak	45°46′20″N 02°57′57″E 1465 m asl	23/06/2016 – 21/09/2016	12	3.04 × 10^5^ ± 4.65 × 10^5 a^	4.82 × 10^3^ ± 1.03 × 10^4 a’e’^	5.77 × 10^2^ ± 1.41 × 10^2 a^	3.70 × 10^2^ ± 1.21 × 10^2 a’b’^
PDM	Pic-du-Midi, France	Terrestrial, high-altitude mountain peak	42°56′11″N 00°08′34″E 2876 m asl	20/06/2016 – 04/10/2016	13	1.51 × 10^5^ ± 1.27 × 10^5 a^	6.40 × 10^3^ ± 5.86 × 10^3 a’d’e’^	5.78 × 10^2^ ± 1.48 × 10^2 a^	5.14 × 10^2^ ± 1.49 × 10^2 b’^
CHC	Chacaltaya, Bolivia	Terrestrial, high-altitude mountain peak	16°20′47″S 68°07′44″W 5380 m asl	27/06/2016 – 11/11/2016	16	1.62 × 10^5^ ± 1.35 × 10^5 a^	1.05 × 10^3^ ± 1.02 × 10^3 e’^	6.63 × 10^2^ ± 2.05 × 10^2 a^	3.59 × 10^2^ ± 7.98 × 10^1 a’ b’^
NAM	Namco, China	Terrestrial, high-altitude plateau	30°46′44″N 90°59′31″E 4730 m asl	16/05/2017 – 14/09/2017	9	3.56 × 10^6^ ± 3.01 × 10^6 c^	4.97 × 10^3^ ± 3.44 × 10^3 a’d’e’^	6.67 × 10^2^ ± 7.48 × 10^1 a^	4.00 × 10^2^ ± 9.08 × 10^1 a’ b’^
STP	Storm-Peak Laboratory, USA	Terrestrial, high-altitude mountain peak	40°27′18″N 106°44′38″E 3220 m asl	11/07/2017 – 04/09/2017	7	1.63 × 10^6^ ± 1.15 × 10^6 ac^	8.78 × 10^4^ ± 6.49 × 10^4 a’d’^	6.62 × 10^2^ ± 1.18 × 10^2 a^	3.52 × 10^2^ ± 2.21 × 10^2 a’ b’^

Reference letters indicate the group membership based on Tukey’s HSD post hoc tests. The environment type, coordinates, elevation from sea level, collection start and end per site and the number of samples collected per site are shown. The 16S rRNA gene qPCR data was previously published in *Dommergue et al., (2019)*^27^.

At all sampling sites, meteorological parameters (*i.e*. wind speed and direction, temperature and relative humidity) were collected every hour. Meteorological data were used to produce wind roses using the openair R package^28^. For each sample, backward trajectories of the air masses were calculated over 3 days (maximum height from sea level: 1 km) using HYSPLIT^29^ and plotted on geographical maps using the openair R package.

We extracted DNA from 3 circular pieces (punches) from the quartz fiber filters (diameter of one punch: 38 mm) using the DNeasy PowerWater kit with the following modifications as detailed in *Dommergue et al*., (2019)^27^. During the cell lysis, we heated at 65°c the PowerBead tube containing the 3 punches and the pre-heated lysis solution during one hour after a 10-min vortex treatment at maximum speed. We then centrifuged the mixture at 1000 rcf during 4 min to separate the filter debris from the lysate using a syringe. From this step, we continued the extraction following the DNeasy PowerWater protocol. DNA concentration was measured using the High Sensitive Qubit Fluorometric Quantification (Thermo Fisher Scientific) then DNA was stored at −20 °C. Real-time qPCR analyses on the 16S rRNA (data previously published in *Dommergue et al., (2019)*^27^) and 18S rRNA genes were carried out (regions, primers^30,31^ and protocols in Supplementary Information) to approximate the concentration of bacterial and fungal cells per cubic meter of air. Although bacteria and fungi might have more than one copy of 16S rRNA and 18S rRNA gene per genome, respectively, attempts to correct for metagenomics datasets are unproductive^32^. Microbial community structure was obtained using MiSeq Illumina amplicon sequencing of the bacterial V3-V4 region of the 16S rRNA gene and the fungal ITS2 region. The library preparation protocol, read quality filtering and taxonomic annotation of every sequence using RDP Classifier^33^ are detailed in Supplementary Information. RDP classifier was used in part to avoid errors due to sequence clustering. The raw read number per sample and the percentage of sequences annotated using RDP Classifier for both the 16S rRNA gene and ITS sequencings are presented in Supplementary Table S1.

All graphical and multivariate statistical analyses were carried out in the R environment, using the vegan^34^, pvclust^35^, ade4^36^ and Hmisc^37^ R packages. The raw abundances of the bacterial genera or fungal species were transformed in relative abundances to counter the heterogeneity in the number of sequences per sample and then standardized using Hellinger’s transformation. Chao1 estimations of the richness for both bacterial and fungal communities were calculated and averaged for each site. MODIS (Moderate resolution imaging spectroradiometer) land cover approach (5′ × 5′ resolution)^38,39^ was used to quantify landscapes in a diameter range of the sampling sites (50, 100 and 300 km). The perimeter of 50 km was chosen because the landscapes were best correlated to airborne microbial community structures. The different MODIS land covers are described in Supplementary Table S2. We weighted these relative surfaces by their associated bacterial cell concentration reported by *Burrows et al., (2009)*^40,41^ (Supplementary Table S3) to predict the relative contribution of each landscape to the aerial emission of bacterial cells.

Hierarchical clustering analyses (average method) were carried out on either the Bray-Curtis dissimilarity matrix (bacterial and fungal community structure) or the Euclidean distance matrix (PM10 chemistry, landscapes and the relative contributions of the landscapes). A Mantel test was used to evaluate the similarities in the distribution of the samples in the different data sets. Distance-based redundancy analyses (RDA) were carried out to evaluate the part of the variance between the samples based on the microbial community structure explained by chemistry. Prior to the cluster analysis based of the chemical dataset, chemical concentrations were log10-transformed to approach a Gaussian distribution. SIMPER analysis was used to identify the major bacterial or fungal contributors to the difference between the detected groups.

Spearman correlations were calculated to test the correlation between microbial abundance and richness and quantitative environmental factors like chemical concentrations or meteorological parameters. ANOVAs were used to test the influence of qualitative factors such as localization on both bacterial and fungal abundance and richness and TukeyHSD tests to identify which group had a significantly different mean. To compare the temporal variability in microbial communities at each site, we interpreted both the Bray-Curtis dissimilarity value averaged per site and the associated standard deviation. We calculated a new statistic, *i.e*. a similarity index, as follows. We averaged the values of dissimilarity obtained from the Bray-Curtis matrix for each pair of samples from the same site. Then we subtracted these values from 1 to get similarity values and finally we divided the similarity values by the standard deviation $(\frac{1\,-\,Bray\,Curtis\,dissimilarity\,value\,averaged\,per\,site}{Bray\,Curtis\,standard\,deviation})$. The higher is the similarity in microbial community structure between the samples, the higher is the similarity index. A multiple linear regression model was used to test the influence of wind and surrounding landscape characteristics (number of different landscapes and landscape evenness) of the sites on the temporal variability of both the fungal and bacterial community structure (evaluated by the similarity index). Pielou’s evenness was used to determine how similar the different relative surfaces of each landscape surrounding the sites were. The temporal variability of meteorological parameters was calculated both within weekly samples (average variance of hourly data for one week) and between weekly samples of the same site (standard deviation of the above average variance over weeks).

## Results

### Geographical distribution of airborne microbial communities

Airborne microbial concentrations varied between 9.2 × 10^1^ to 1.3 × 10^8^ cells per cubic meter of air for bacteria and from not detectable (< 2 × 10°) to 1.9 × 10^5^ cells per cubic meter of air for fungi. A high correlation was observed between the bacterial and the fungal concentrations from all the sites and all sampling times (*N* = 95, *R* = 0.85, *P* = 2.2 × 10^−16^). The average bacterial and fungal concentrations per site varied from 7.3 × 10^2^ to 3.6 × 10^6^ and from 5.2 × 10° to 8.8 × 10^4^ cells per cubic meter of air, respectively, and were different between the sites (*P* = 2.2 × 10^−11^ and *P* = 5.7 × 10^−15^ for bacteria and fungi, respectively; Table 1). The polar site Station-Nord and the high altitude plateau site of Namco had the lowest and highest average bacterial concentration, respectively. The highest average fungal concentration was observed in atmospheric samples from the urban site of Grenoble and the mountain site of Storm-Peak, while the lowest was from Station-Nord (Table 1).

**Figure 2:**
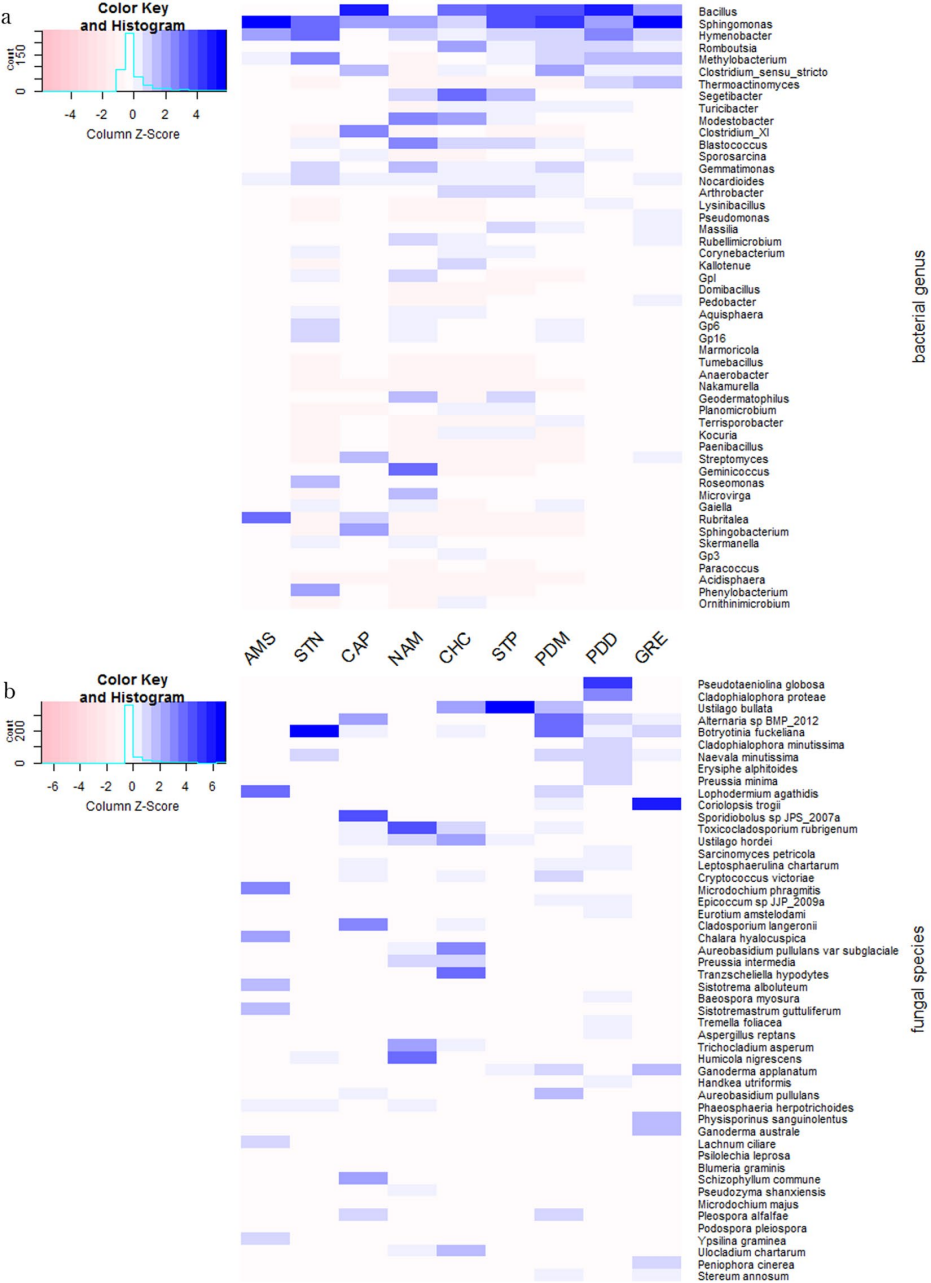
Heatmaps of the relative abundances (the relative abundances are centered and scaled) of the fifty most abundant bacterial genera (**a**) and fungal species (**b**) in the dataset. The fifty bacterial genera and fungal species are in order of decreasing relative abundance from top to bottom.

The most abundant bacterial genera overall for all the samples were *Bacillus* (8.23%), *Sphingomonas* (5.62%), *Hymenobacter* (4.32%), *Romboutsia* (2.77%), *Methylobacterium* (2.63%), and *Clostridium* (2.18%) (a list of the highest 50 genera is shown in Supplementary Table S4), although their relative contribution to each site varied. A heatmap of the relative abundances of the fifty most abundant bacterial genera in each site is represented in Fig. 2. For example, *Bacillus* averaged 15.63% relative abundance in Puy-de-Dôme samples, but only 0.03% in the marine Amsterdam-Island samples. Average bacterial Chao1 richness estimations varied between 577 + − 141 (Puy-de-Dôme) and 217 + − 76 (Station-Nord). They did not differ significantly (*P* > 0.05) between sites with the exception of Station-Nord which showed the lowest Chao1 value (*P* = 4.9 × 10^−11^) (Table 1). The most abundant fungal species in the whole dataset were *Pseudotaeniolina globosa* (5.44%), *Cladophialophora proteae* (3.67%), *Ustilago bullata* (3.22%), *Alternaria* sp (2.60%), and *Botryotinia fuckeliana* (*Botrytis cinerea*) (2.47%) (a list of the highest 50 species is shown in Supplementary Table S5), although their relative contribution to each site varied. A heatmap of the relative abundances of the fifty most abundant fungal species in each site is represented in Fig. 2. For example, *Pseudotaeniolina globosa* averaged 11.47% relative abundance in Puy-de-Dôme samples, but less than 0.01% in Station-Nord and Storm-Peak samples. Average fungal Chao1 richness estimations were different between the sites (*P* = 9.5 × 10^−14^) with the highest value from Grenoble (742 +/− 106) and lowest values from Cape-Point (440 +/−152), Amsterdam-Island (225 +/− 45) and Station-Nord (104 +/− 51). The bacterial and fungal Chao1 richness estimations correlated with the bacterial and fungal concentrations, respectively (*N* = 81, *R* = 0.61, *P* = 1.0 × 10^−9^ and *N* = 79, *R* = 0.58, *P* = 2.0 × 10^−8^, respectively).

**Figure 3:**
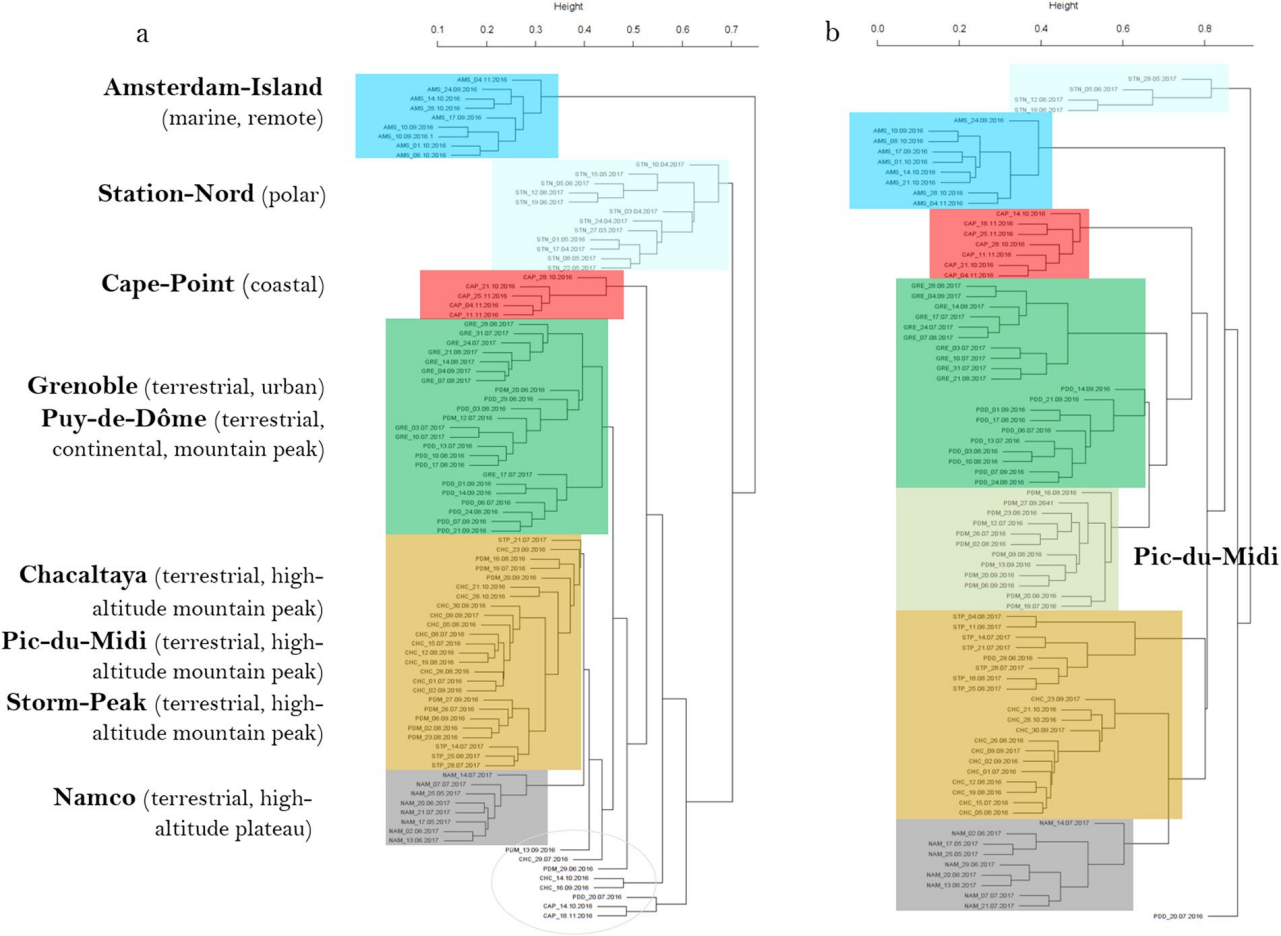
Hierarchical cluster analysis (average method) of the Bray-Curtis dissimilarity matrices based on the (**a**) V3-V4 region of the 16S rRNA gene (genus level) and (**b**) ITS region (species level). Colored rectangles correspond to samples of the same site or group of sites stated beside the rectangles. Outlier samples (samples which were outside their expected group) are circled in grey. Samples are named as follows: site_date.of. sampling.

**Figure 4:**
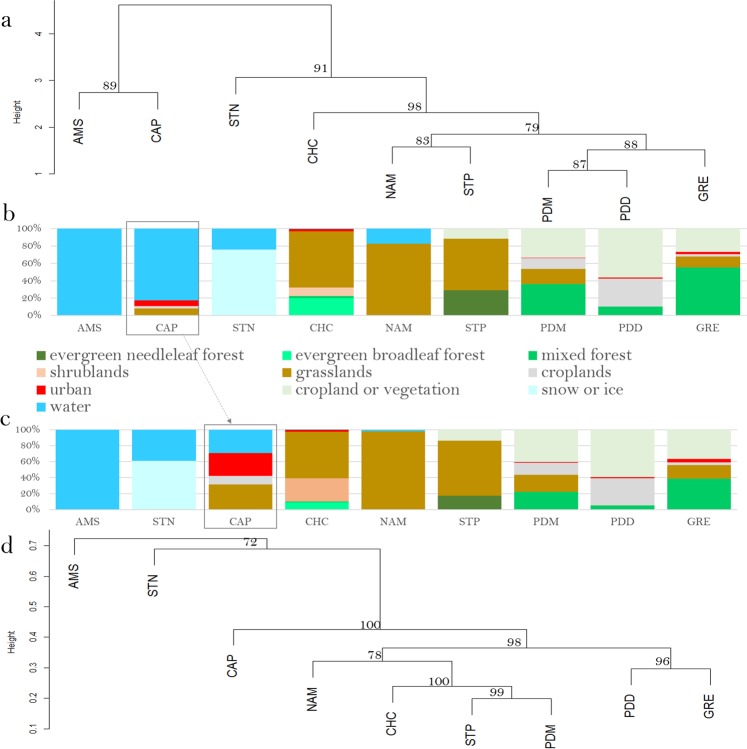
Distribution of the sites based on the different data sets (bacterial community structure, PM10 chemistry, landscapes, relative contributions of the different landscapes in the aerial emission of bacterial cells). (**a**) Hierarchical cluster analysis (average method) on the Euclidean distance matrix based on the PM10 chemistry. (**b**) Relative surfaces of the different landscapes surrounding the sites (perimeter of 50 km) based on the MODIS land cover approach. (**c**) Relative contributions of the different landscapes in the aerial emission of bacterial cells (based on the study of *Burrows et al., (2009)*^40^). (**d**) Hierarchical cluster analysis (average method) on the Bray-Curtis dissimilarity matrix based on the bacterial community structure (genus level). Bootstrap values in percentage are indicated over each node on both cluster analyses.

The different temporal samples grouped mainly by their site of origin using the hierarchical cluster analyses based on both their bacterial and fungal community structure (Fig. 3; for an expanded view see Supplementary Fig. S1) and using the bacterial and fungal community profiles averaged over the multiple samples per site (Fig. 4 and Supplementary Fig. S2). The sites grouped into three distinct clusters on both the bacterial and fungal-based trees: one cluster included the marine site Amsterdam-Island, the second the polar site Station-Nord and the third one included all the terrestrial and non-polar sites including the coastal site Cape-Point (Fig. 4 and Supplementary Fig. S2). The different sites or groups of sites were characterized by bacterial genera and fungal species known to be associated to the environment type of the site in most cases (Supplementary Table S6). The temporal variability of the composition of the bacterial and fungal communities at each site was different between the sites (*P* < 2 × 10^−16^ for both bacterial and fungal communities; Table 2). Temporal variations in the bacterial community structure were not correlated to the variations in the fungal community structure (*R* = 0.13, *P* = 0.74). The highest temporal variability of the composition of the bacterial communities was observed in Cape-Point (similarity index of 3.9), Chacaltaya (5.4) and Station-Nord (5.5), while the lowest temporal variability was observed in Amsterdam-Island (17.5), Storm-Peak (17.4) and Namco (20.8). The highest temporal variability of the composition of the fungal communities was observed in Station-Nord (similarity index of 1.6), Puy-de-Dôme (2.95) and Storm-Peak (4.7), while the lowest temporal variability was observed in Amsterdam-Island (9.3), Cape-Point (9.3) and Pic-du-Midi (9.6; Table 2). We observed eight “outlier” samples (*i.e*. samples which did not group with the other samples of their respective site) out of eighty-two samples using the hierarchical cluster analysis based on the bacterial community structure of the individual samples (Fig. 3). Only one outlier was observed using the hierarchical cluster analysis based on the fungal community structure (Fig. 3).

**Table 2:** Temporal variability of the microbial community structure and meteorological conditions at each site. The similarity index was calculated as following: (1 – Bray-Curtis dissimilarity value averaged per site)/standard deviation.

Site	Bacterial community structure similarity between weeks (similarity index)	Fungal community structure similarity between weeks (similarity index)	Number of different landscapes within a 50 km perimeter	Landscape evenness (Pielou’s evenness)	Maximum wind speed (m/s)	Wind direction variability within weeks (degree)	Wind direction variability between weeks (degree)	Relative humidity variability within weeks (%)	Relative humidity variability between weeks (%)	Temperature variability within weeks (°C)	Temperature variability between weeks (°C)
AMS	17.5	9.3	1	1	22	62.3	29.8	10.7	4	1.2	1
CAP	3.9	9.3	4	0.47	26.7	79.5	33.4	11.8	5.6	2.2	1
GRE	12.5	7.6	5	0.65	9.6	94.5	15.3	17.8	5.8	4.9	2.9
STN	5.5	1.6	2	0.80	12.1	81.9	28.6	7.7	5.3	2.7	19.4
PDD	6.81	2.95	4	0,71	31.1	91	34.6	17.6	11.1	3.92	23
PDM	9.6	9.6	4	0.76	32.5	81.8	32.4	20.3	13.4	2.9	3.3
CHC	5.4	7.2	6	0.59	20.1	85.3	47.6	21.6	22.4	1.2	1.2
NAM	20.8	5.3	2	0.66	6.1	29.9	23.2	8.2	6.9	1.2	2.3
STP	17.4	4.7	3	0.84	13	111.1	37	19.5	10.1	3	2.1

The maximum wind speed (m/s) per site, the variability of the wind direction (degree), relative humidity (%) and temperature (°C) within a week and between the weeks, the number of different landscapes within a perimeter of 50 km and the landscape evenness are shown.

### Potential factors driving chemistry and microbiology

Samples from the same site tended to group together based on the PM10 chemistry using hierarchical cluster analysis (Supplementary Fig. S3). Therefore, we calculated the average chemical profile for each site and redid the cluster analysis (Fig. 4). The sites were separated into three main clusters using a hierarchical cluster analysis based on the average chemical profiles (Fig. 4): the first cluster (Amsterdam-Island, Cape-Point) was characterized by high relative concentrations of sea salts (Cl, Na), the second (Puy-de-Dôme, Grenoble, Pic-du-Midi, Chacaltaya, Namco, Storm-Peak) by higher concentrations of organic carbon, polyols and sugars, and the third (Station-Nord) by very low relative concentrations of polyols and sugars and lower relative concentrations of sea salts compared to the first cluster (Supplementary Table S7). The PM10 chemistry was correlated to the bacterial and fungal community structure averaged over the multiple samples per site (Mantel test *R* = 0.79 *P* = 0.007 and *R* = 0.68 and *P* = 0.02, respectively) (see RDA figures in Supplementary Fig. S4). The differences between the marine site (Amsterdam-Island), coastal site (Cape-Point), polar site (Station-Nord) and terrestrial sites in terms of PM10 chemistry and microbial communities might create this correlation. Thus, we looked at the correlation using only the terrestrial sites and the correlation between the PM10 chemistry and the bacterial and fungal community structure was weaker (*R* = 0.16, *P* = 0.33 and *R* = 0.51, *P* = 0.03 for bacterial and fungal community structure, respectively).

The different landscapes (e.g., forest, cropland, ocean, etc.) surrounding the sites were identified using the MODIS land cover approach (Fig. 4). We weighted the relative surface of the different landscapes by their associated bacterial cell concentration reported by *Burrows et al., (2009*^)40,41^ to predict the relative contribution of each landscape to the aerial emission of bacterial cells at each site (Fig. 4). Terrestrial sites grouped together based on the high contribution of their terrestrial landscape to the aerial emission of bacterial cells. The average PM10 chemical profile for the different sites was only correlated to the relative surfaces of the different landscapes when considering all the sites (*R* = 0.77, *P* = 0.001), but not when considering only the terrestrial sites (*R* = 0.32, *P* = 0.1). Both the bacterial and fungal communities were correlated to the landscapes over all the sites (*R* = 0.80, *P* = 0.001; *R* = 0.64, *P* = 0.003, respectively). However, unlike the chemistry, the bacterial community structure averaged over the multiple samples was correlated to the relative surfaces of the different landscapes for the terrestrial sites (*R* = 0.65, *P* = 0.004). The same was observed for the fungal community structure averaged over the multiple temporal samples which was also correlated to the landscapes of the terrestrial sites (*R* = 0.47, *P* = 0.05). Although the bacterial community distribution was correlated to the fungal community distribution (*R* = 0.82, *P* = 0.006), some differences were observed. The mountain sites (Namco, Chacaltaya, Storm-Peak and Pic-du-Midi) grouped together in the cluster analysis based on their bacterial community structure, while the sites characterized by croplands as one of their surrounding landscapes (Grenoble, Pic-du-Midi, Puy-de-Dôme and Cape-Point) grouped together in the cluster analysis based on the fungal community diversity (Fig. 4 and Supplementary Fig. S2 for the cluster analysis based on the bacterial and fungal diversity, respectively).

Meteorological characteristics (wind speed and hourly variations in wind direction, relative humidity, and temperature over one week and between weeks) were different between the sites (Table 2). Puy-de-Dôme, Pic-du-Midi and Cape-Point showed episodes of high wind speeds (between 26 and 32 m/s), while the wind speed did not reach 10 m/s in Grenoble and Namco. The wind direction varied within the weeks of sampling and also between the multiple weekly samples per site, and these variations were not necessarily correlated. For example, the wind direction in Grenoble varied continually within the weeks (mean variability within weeks: 94.5°), and this variability did not change much between the weeks (mean variability between weeks: 34.6°). The wind direction in Chacaltaya changed less within the weeks (35.3°) than between the weeks (47.6°). The different temporal variability of both the wind speed and direction led to different wind roses and backward air mass trajectories with different directions and lengths at each site (Supplementary Figs S5,S6). The relative humidity and temperature also showed a different temporal (within and between weeks) variability depending on the site (Table 2). Multiple linear regressions between the level of temporal variability of both the bacterial and fungal community structures at each site and the meteorological and surrounding landscape characteristics (number of different landscapes and landscape evenness) were calculated. For the variation of bacterial composition over time, the regression included the wind direction variability within and between weeks, the temperature variability between weeks and the landscape evenness (*R* squared = 0.93, adjusted *R* squared = 0.82, *P* = 0.06). For the variation of fungal composition over time, the wind speed and temperature variability between weeks were the best parameters (*R* squared = 0.87, adjusted *R* squared = 0.83, *P* = 0.002) (Supplementary Table S8 and Fig. S7).

## Discussion

Airborne particulate matter (PM) is thought to undergo both short and long-range transport depending mainly on particle size^42–44^. Airborne microbial cells might exist both as free cells and in association to PM in the atmosphere^45–47^. This means that the sources, transport duration and deposition processes could be different between PM and microbial cells, especially if their size distribution is different. Furthermore, the potential transformation processes that they might undergo during atmospheric transport are likely to be different. While photochemistry and gas condensation will change the chemical composition and size distribution of PM^48^, selective processes associated mainly to UV radiation, desiccation and cold temperatures could lead to the death, sporulation and genetic mutations of microbial cells, although the occurrence of these processes in the atmosphere have been rarely explored^17,49^. Thus, a correlation between airborne microbial community structure and the chemical composition of PM is unlikely due to their expected differential fate during transportation in the atmosphere. So while PM10 chemistry correlated to both the bacterial (*R* = 0.79, *P* = 0.007) and fungal (*R* = 0.68, *P* = 0.02) community structures over all the sites, the correlation was considerably less for the terrestrial sites for bacteria (*R* = 0.16, *P* = 0.33) and fungi (*R* = 0.51, *P* = 0.03). The strong correlation observed when considering all the sites might be more indicative of strong differences between the different ecosystems (marine, polar, coastal and terrestrial) in terms of chemistry and microbial communities than a similar behavior and/or driving processes of the PM10 and microbial cell distribution.

This weak correlation between PM10 chemistry and airborne bacterial communities over the terrestrial sites has been observed previously^1,18^, but certain chemicals might affect specific microbial populations without influencing the overall correlation. For example, methylsulfonic acid (MSA) was correlated to the genus *Acholeplasma* (*R* = 0.88, *P* = 1.7 × 10^−3^). Certain polyol concentrations were correlated to the relative abundance of specific fungi (*e.g*., mannitol-arabitol and the species *Zalerion arboricola R* = 0.85, *P* = 3.9 × 10^−3^). Polyols can be major components of fungal biomass and have been proposed as a suitable marker for fungal spores^50^. We currently do not know whether these chemistry-microbial genera/species correlations are due to similar sources for both PM10 and microorganisms or that the chemistry is selecting for these microorganisms during atmospheric transportation.

The similarity of the sites based on the PM10 chemistry globally matched the similarity of the sites based on the characteristics of the landscapes surrounding the sites (Fig. 4). However, since the correlation between the chemistry and the characteristics of the surrounding area was not very high when considering only the terrestrial sites (*R* = 0.32, *P* = 0.1), distant sources and/or photochemical transformations (formation of secondary aerosols) likely also contribute to the PM10 chemistry distribution. A strong correlation was observed between both airborne bacterial (*R* = 0.80, *P* = 0.001 with all the sites; *R* = 0.65, *P* = 0.004 with only the terrestrial sites) and fungal community structure (*R* = 0.64, *P* = 0.003 with all the sites; *R* = 0.47, *P* = 0.05 with only the terrestrial sites) and the landscapes (Fig. 4 and Supplementary Fig. S2). This correlation illustrates the important contribution of the local landscapes to the geographical distribution of airborne microorganisms. The landscapes with the most impact on the distribution of atmospheric microorganisms between the sites were ice, water, forest, grassland and cropland. These landscapes might emit distinct communities as compared to the others such as urban landscapes which did not seem to significantly contribute to the observed geographical microbial distribution of the sites. Regional environmental factors, and specifically, a combination of climate and soil characteristics (but no effect from urbanization) have been reported to correlate to the geographical distribution of dust-associated microbial communities^10^. Reports of other smaller-scale studies^2,13,16,18^ suggested a strong influence of the local sources in the spatial distribution of airborne microbial communities. Airborne microbial community structure at the coastal site Cape-Point was more similar to that for terrestrial sites than those from marine or polar sites. This might reflect that terrestrial landscapes contribute more biomass to airborne microbial communities than oceanic landscapes, as was proposed by *Burrows et al., (2009)*^40,41^.

Our airborne bacterial concentrations were mainly correlated to the surrounding landscape. We observed the highest atmospheric bacterial concentrations at the grassland sites (Namco, Storm-Peak) and urban and cropland sites (Grenoble, Puy-de-Dôme), followed by the coastal site (Cape-Point), marine site (Amsterdam-Island) and polar site (Station-Nord). We did not observe the highest average concentration of bacteria at the most urban site (Grenoble) as expected based on the study of *Burrows et al., (2009)*^40,41^, although Grenoble might not be as industrial as others reported in the literature^1,11^. Instead, the highest average concentration of bacteria was observed at Namco, which is a remote high-altitude and semi-arid grassland site (grassland covers >80% of the surrounding landscape over 50 km of diameter). Pic-du-Midi (cropland/vegetation) and Chacaltaya (grassland), which are two high-altitude mountain peaks, had relatively low average bacterial concentrations that were comparable to the average bacterial concentration of the coastal site Cape-Point. The elevation and steep slopes of Pic-du-Midi and Chacaltaya could explain the relatively low airborne bacterial concentrations, since they might limit upward migration of aerosolized bacteria from land surfaces to peaks. Another explanation could be that Chacaltaya and Pic-du-Midi aerosols were sampled mainly from the free-troposphere that has fewer microorganisms than the planetary boundary layer. At the Namco site, the calm meteorological conditions (low wind speed, low relative humidity and a low precipitation rate), in association with the high dust content of the surrounding landscape could explain its high bacterial concentrations. The effect of meteorological conditions on airborne microbial concentrations have been investigated previously^51–53^, but the wind speed, for example, could lead to either an increase or a decrease in the bacterial concentration depending on its direction, speed and site characteristics.

We hypothesized that meteorological conditions could have an influence on the temporal variation of airborne microbial community structure by affecting the relative inputs of different microbial populations from different surrounding landscapes. Our data showed that the temporal variability of microbial community structure was significantly different between the sites and could be correlated (*R* > 0.80) with wind condition variability, temperature variability and/or landscape characteristics (number of different landscapes and landscape evenness). Wind conditions and temperature are both known to affect the aerosolization process^54,55^. The wind speed was an important factor explaining fungal variability as different wind speeds might lift up different fungal spore sizes and weights^56,57^. The characteristics of the surrounding landscape (number of different landscapes and landscape evenness) were also important factors determining the temporal variability of airborne microbial communities. Namco and Amsterdam-Island had relatively low temporal variability of airborne bacterial community structure in concordance with their relatively monospecific landscapes, grassland and oceanic, respectively. Although Amsterdam-Island site is characterized by high wind speed, the presence of an oceanic surface over a large perimeter around the site imposed a predominance of homogeneous atmospheric microbial communities. Consequently, wind speed and direction could be of importance when the surrounding landscape is diverse. Although Grenoble has five different surrounding landscapes, the low wind speed (<9.6 m/s) in Grenoble would lead to a relatively low temporal variability of the airborne bacterial community structure. Conversely, the association of different surrounding landscapes and a medium (between 12 m/s and 13 m/s) or high wind speed (>22 m/s), as found at the other sites would increase the variability of the composition of bacterial communities over time as a function of the wind speed, wind direction variability and the number of different landscapes. We think that the changes in the composition of airborne bacterial communities might be due to the combined effect of changes in landscapes and local meteorological conditions. Changes in seasons will likely affect landscapes and meteorological conditions and, therefore, influence microbial community composition^13–16,18,58^.

## Conclusion

This is the first worldwide-scale study investigating airborne microbial communities at diverse sites in terms of latitudinal position, type of ecosystem, surrounding landscapes and local meteorological conditions. We also investigated the atmospheric particulate matter, surrounding landscapes via the MODIS land cover approach, and local meteorology to assess their role in defining the atmospheric microbial communities. We observed that airborne microbial communities were correlated to the surrounding landscapes, although some (minor fraction) of the microbial cells might travel over long distances. While two sites sharing similar surrounding landscapes will likely get a similar airborne microbial profile, different local meteorological conditions will control the stability of this microbial profile through time. In the context of global warming and land use changes atmospheric microbial (including viruses) communities should be continually monitored around our planet.

## Supplementary information

Supplementary Information

## Data Availability

Sequences reported in this paper have been deposited in ftp://ftp-adn.ec-lyon.fr/aerobiology_amplicon_INHALE/. A file has been attached explaining the correspondence between file names and samples.
