# Author Correction: Global airborne microbial communities controlled by surrounding landscapes and wind conditions

**DOI:** 10.1038/s41598-020-59138-5

**Published:** 2020-02-06

**Authors:** Romie Tignat-Perrier, Aurélien Dommergue, Alban Thollot, Christoph Keuschnig, Olivier Magand, Timothy M. Vogel, Catherine Larose

**Affiliations:** 1 Institut des Géosciences de l’Environnement, Univ Grenoble Alpes, CNRS, IRD, Grenoble INP, Grenoble, France; 2 Environmental Microbial Genomics, Laboratoire Ampère, Ecole Centrale de Lyon, Université de Lyon, Ecully, France

Correction to: *Scientific Reports* 10.1038/s41598-019-51073-4, published online 08 October 2019

This Article contains errors in Reference 55, which was incorrectly given as:

Pietsch, R. B., David, R. F., Marr, L. C., Vinatzer, B. III. & D. G. S. Aerosolization of Two Strains (Ice+ and Ice−) of Pseudomonas syringae in a Collison Nebulizer at Different Temperatures. *Aerosol Science and Technology* **49**, 159–166 (2015).

The correct reference is listed below:

Pietsch, R. B., David, R. F., Marr, L. C., Vinatzer, B. & Schmale, D. G. III. Aerosolization of Two Strains (Ice+ and Ice−) of Pseudomonas syringae in a Collison Nebulizer at Different Temperatures. *Aerosol Science and Technology* **49**, 159–166 (2015).

Furthermore, the Article contains errors in Table 1, where the heading for column 7 should read ‘16S rRNA gene copies/m^3^’ not ‘16S rRNA gene copies/m’ and the heading for column 8 should read ‘18S rRNA gene copies/m^3^’ not ‘18S rRNA gene copies/m’.

