# Supplementary material for: Global airborne microbial communities controlled by surrounding landscapes and wind conditions
Source: Sci Rep. 2019 Oct 8;9:14441. doi: 10.1038/s41598-019-51073-4 (PMC6783533; doi:10.1038/s41598-019-51073-4)
Supplement: Supplementary file 1 — Supplementary Information [file 41598_2019_51073_MOESM1_ESM.docx]

Global airborne microbial communities controlled by surrounding landscapes and wind conditions

Romie Tignat-Perrier^1,2*^, Aurélien Dommergue^1^, Alban Thollot^1^, Christoph Keuschnig^2^, Olivier Magand^1^, Timothy M. Vogel^2^, Catherine Larose^2^

^1^Institut des Géosciences de l’Environnement, Université Grenoble Alpes, CNRS, IRD, Grenoble INP, Grenoble, France

^2^Environmental Microbial Genomics, Laboratoire Ampère, École Centrale de Lyon, Université de Lyon, Écully, France

[*romie.tignat@univ-grenoble-alpes.fr](mailto:*romie.tignat@univ-grenoble-alpes.fr)

Supplementary Information Text

**Material and Methods**

*Real-Time qPCR analyses*

***16S rRNA gene qPCR.*** The bacterial cell concentration was approximated by the number of *16S rRNA* gene copies per cubic meter of air. The V3 region of the *16S rRNA* gene was amplified using the SensiFast SYBR No-Rox kit (Bioline) and the following primers sequences: Eub 338f 5’-ACTCCTACGGGAGGCAGCAG-3’ as the forward primer and Eub 518r 5’-ATTACCGCGGCTGCTGG-3’ as the reverse primer^1^ on a Rotorgene 3000 machine (Qiagen). The reaction mixture of 20µL contained 10µL of SYBR master mix, 2µL of DNA and RNAse-free water to complete the final 20µL volume. The qPCR 2-steps program consisted of an initial step at 95°C for 2min for enzyme activation, then 35 cycles of 5sec at 95°C and 20sec at 60°C hybridization and elongation. A final step was added to obtain a denaturation from 55°C to 95°C with increments of 1°C.s^-1^. The amplicon length was around 200 bp. PCR products obtained from DNA from a pure culture of *Escherichia* *coli* were cloned in a plasmid (pCR™2.1-TOPO® vector, Invitrogen) and used as standard after quantification with the Broad-Range Qubit Fluorometric Quantification (Thermo Fisher Scientific).

***18S rRNA gene qPCR.*** The fungal cell concentration was approximated by the number of *18S rRNA* gene copies per cubic meter of air. The region located at the end of the SSU *18S rRNA* gene, near the ITS1 region, was quantified using the SensiFast SYBR No-Rox kit (Bioline) and the following primers sequences: FR1 5’-AICCATTCAATCGGTAIT-3’ as the forward primer and FF390 5’-CGATAACGAACGAGACCT-3’ as the reverse primer ^2^ on a Rotorgene 3000 machine (Qiagen). The reaction mixture of 20µL contained 10µL of SYBR master mix, 2µL of DNA and RNAse-free water to complete the final 20µL volume. The qPCR 2-steps program consisted of an initial step at 95°C for 5min for enzyme activation, then 35 cycles of 15sec at 95°C and 30sec at 60°C hybridization and elongation. A final step was added to obtain a denaturation from 55°C to 95°C with increments of 1°C.s^-1^. The amplicon length was around 390 bp. PCR products obtained from DNA from a soil sample were cloned in a plasmid (pCR™2.1-TOPO® vector, Invitrogen) and used as standard after quantification with the Broad-Range Qubit Fluorometric Quantification (Thermo Fisher Scientific).

*MiSeq Illumina amplicon sequencing:* ***library preparation.*** The V3-V4 region of the *16S rRNA* gene was amplified using the Platinum Taq Polymerase (ThermoFisher Scientific) using the following primer sequences: 5'-TCGTCGGCAGCGTCAGATGTGTATAAGAGACAGCCTACGGGNGGCWGCAG-3’ as the forward primer sequence, and 5'-TCGTCGGCAGCGTCAGATGTGTATAAGAGACAGCCTACGGGNGGCWGCAG-3’ as the reverse primer sequence. The PCR program used was: 95°C for 3 minutes, 35 cycles of 95°C for 30 seconds, 55°C for 30 seconds and 72°C for 30 seconds, then a final step of 72°C for 5 minutes. The average amplicon size was 550 bp.

To test the reproducibility of the molecular biology analyses, we divided by two (technical duplicates) two samples (AMS_10.09.2016 and PDD_21.12.2016) and did separately the different analyses from the DNA extraction to the *16s rRNA* gene sequencing.

The ITS2 region was amplified using the Platinum Taq Polymerase (ThermoFisher Scientific) using the following primer sequences: ILL_5.8S_Fun 5' TCGTCGGCAGCGTCAGATGTGTATAAGAGACAGAACTTTYRRCAAYGGATCWCT 3’ as the forward primer sequence, and ILL_ITS4_Fun 5' GTCTCGTGGGCTCGGAGATGTGTATAAGAGACAGAGCCTCCGCTTATTGATATGCTTAART 3’ as the reverse primer sequence ^3^. The PCR program used was: 95°C for 3 minutes, 35 cycles of 95°C for 30 seconds, 56°C for 30 seconds and 72°C for 60 seconds, then a final step of 72°C for 10 minutes. The average amplicon size was 510 bp.

The other steps of the library preparation (amplicon PCR clean-up, index PCR, index PCR clean-up, normalization and pooling) were performed following the Illumina library preparation protocol (“16S Metagenomic Sequencing Library Preparation”). The amplicons were sequenced by a paired-end MiSeq sequencing using the technology V3 (*16S rRNA* gene) and V2 (ITS) of Illumina with 2 x 250 cycles. The adapter sequences were removed by internal Illumina software at the end of the sequencing. Samples under 6000 raw reads were removed from the dataset with the exception of the arctic Station-Nord samples.

***Reads quality filtering and taxonomic annotation.*** The base quality of the reads 1 and reads 2 was controlled (quality filtering using Q20) using tools of the FASTX-Toolkit software (http://hannonlab.cshl.edu/fastx_toolkit/). PANDAseq^3^ was used to assemble the read 1 and the read 2 using the RDP algorithm, a minimum and maximum length of the resulting sequence of 410 bp and 500 bp for *16S rRNA* gene sequencing, and 390 bp and 500 bp for ITS region sequencing, a minimum and maximum overlap length of 20 bp and 100 bp. The resulting sequences were stripped out from the primers and annotated at the genus or species level by RDP Classifier^4^ using the RDP 16srrna and fungallsu databases for *16S rRNA* gene and ITS sequencing, respectively, and an assignment confidence cutoff of 0.6. Sequence analyses were done on the Newton supercalculator of the Ecole Centrale de Lyon. The number of sequences per sample and the percentage of sequences annotated at the genus (bacteria) and species (fungi) level were evaluated using a home-made R script (Supplementary **Table S1**). The sequences annotated as chloroplasts were removed.

Supplementary Information Figures

**Results**


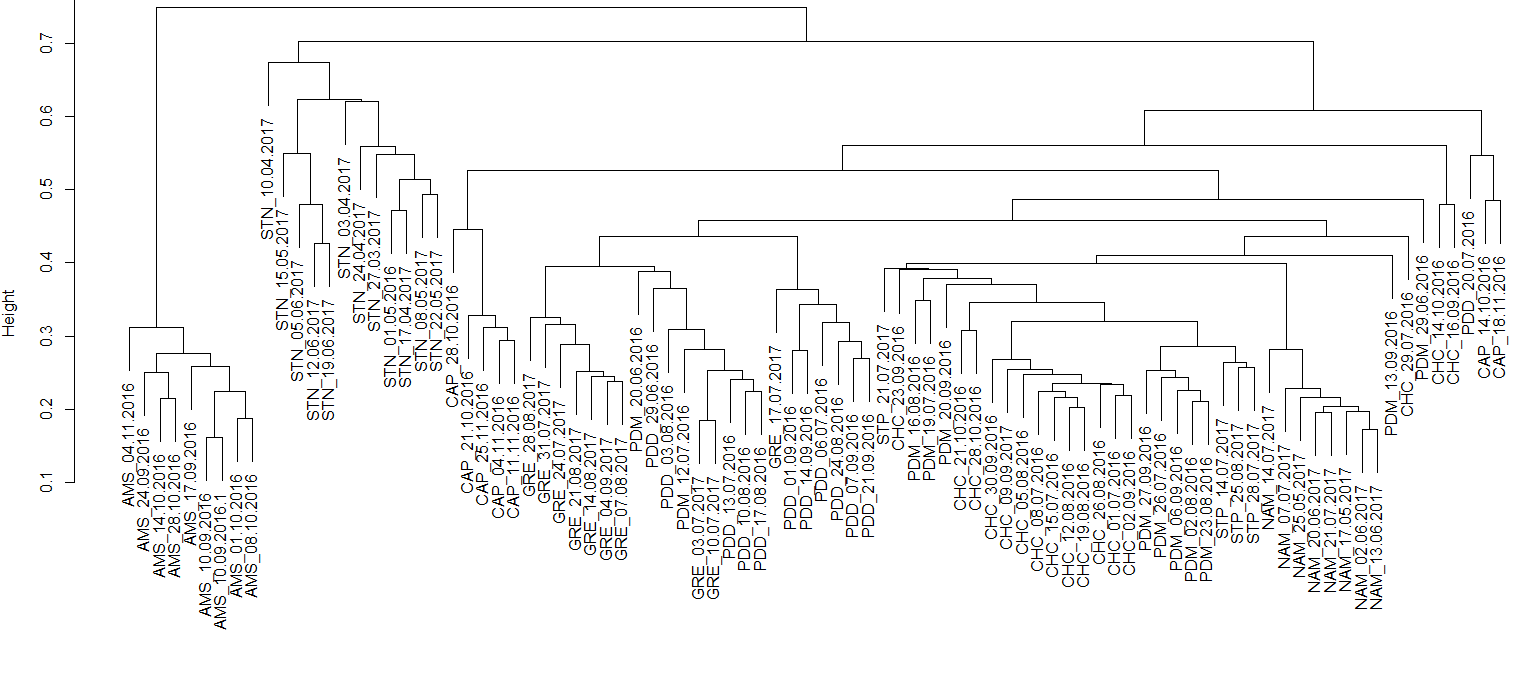

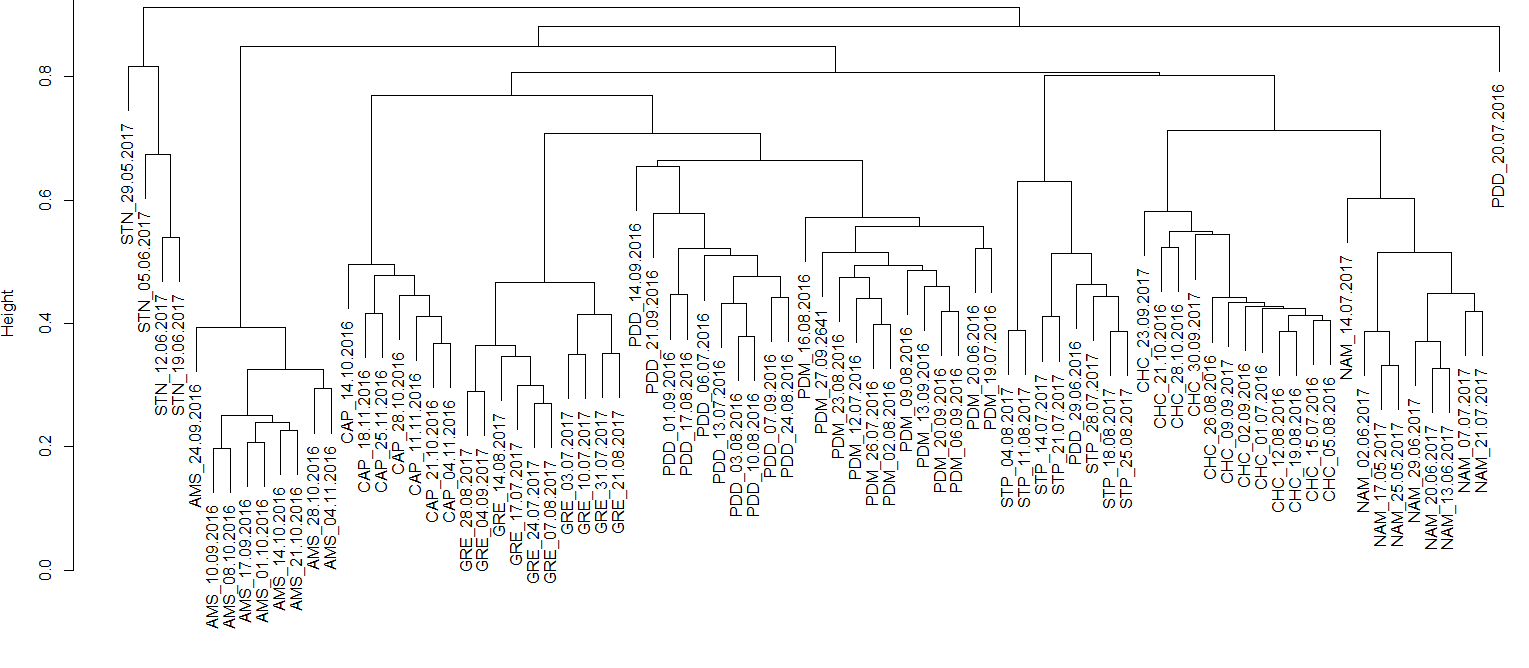


b

a

Supplementary Figure S1. Hierarchical cluster analysis (average method) of the Bray-Curtis dissimilarity matrices based on the V3-V4 region of the *16S rRNA* gene (a) and ITS region (b). Samples are named as follows: site_date.of.sampling.


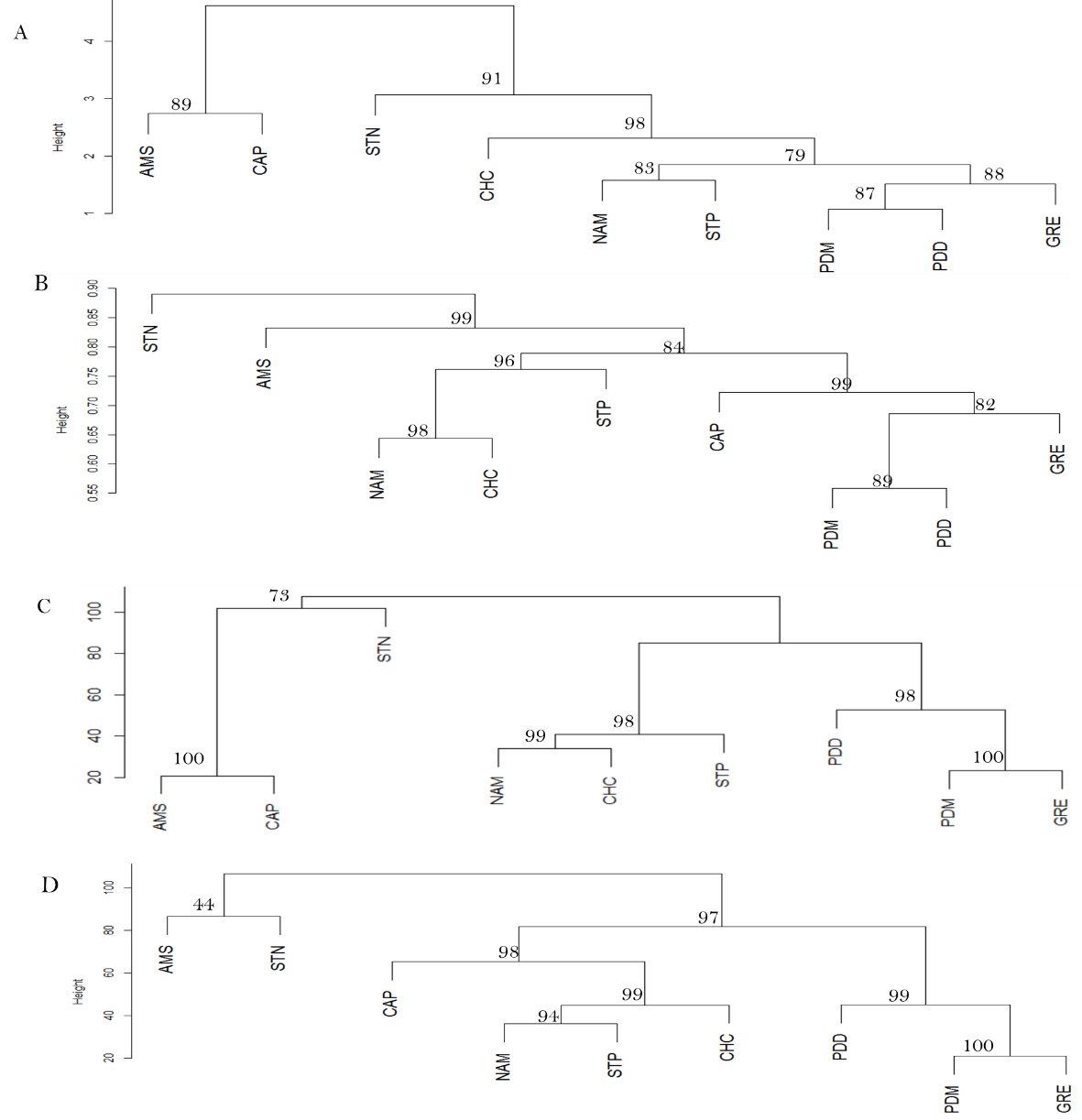


d

b

c

a

Supplementary Figure S2. Distribution of the sites based on their (a) average chemical profile, (b) average fungal community profile, (c) relative surfaces of the different landscapes surrounding the sites, (d) relative contributions of the landscapes in the emission of bacterial cells based on the concentration estimates per landscape reported in *Burrows et al., 2009*^5^.

(a) Hierarchical cluster analysis (average method) on the Euclidean dissimilarity matrix calculated on the average composition in chemical species at each site.

(b) Hierarchical cluster analysis (average method) on the Bray-Curtis dissimilarity matrix calculated on the average composition in fungal species at each site.

(c) Hierarchical cluster analysis (average method) on the Euclidean distance matrix calculated on the relative surfaces of the different landscapes

(d)Hierarchical cluster analysis (average method) on the Euclidean distance matrix calculated on the relative contributions of the different landscapes in the aerial emission of bacterial cells. The expected average bacterial concentrations found above each landscape are based on the study of *Burrows et al., 2009*^5^.

Bootstrap values in percentage are indicated over each node.


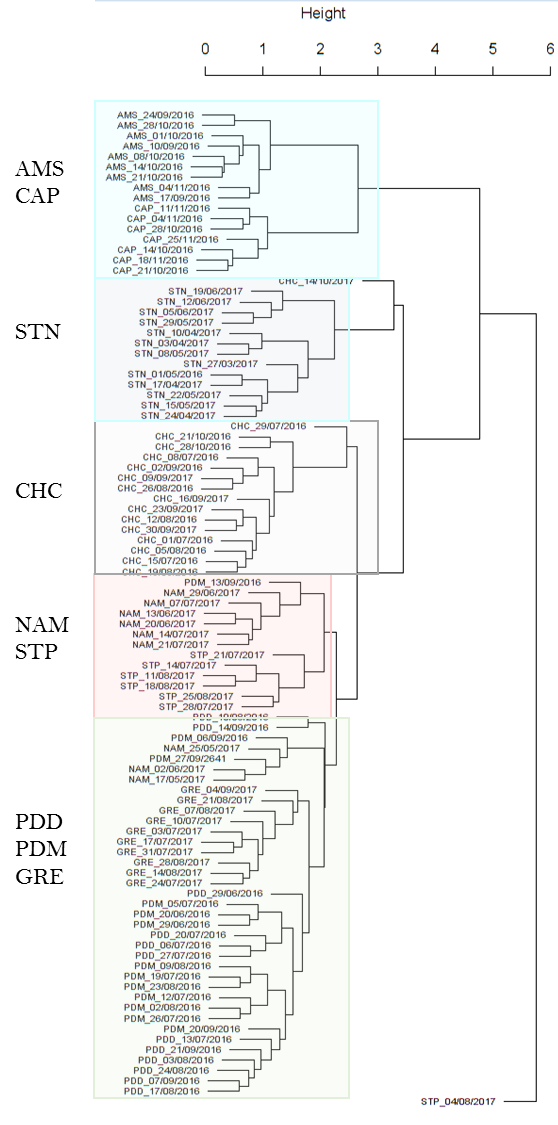


Supplementary Figure S3. Hierarchical cluster analysis (average method) on the Euclidean distance matrix calculated on the PM10 chemistry data. Color rectangles correspond to the samples of the same site or group of sites stated beside the rectangles.


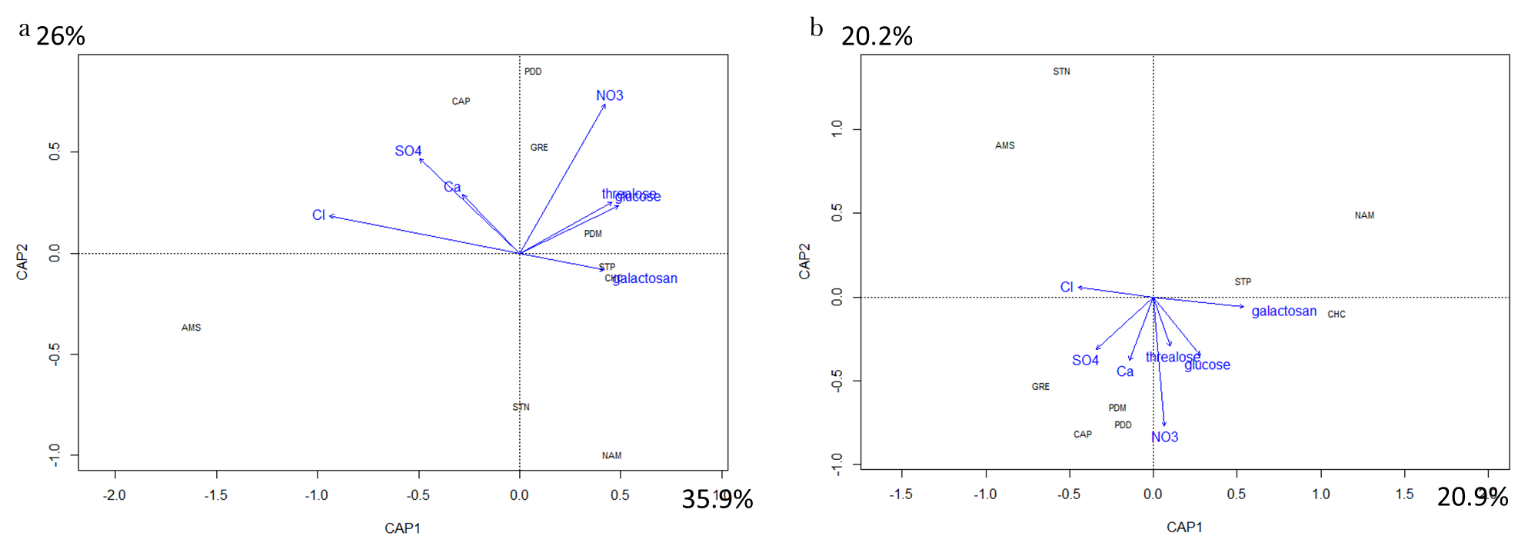


Supplementary Figure S4. Distance-based RDA analyses. Part of variation in the distribution of both the bacterial (a) and fungal (b) community profile averaged by site explained by several chemical variables (non collinear variables having a VIF > 10) on the first two axes.


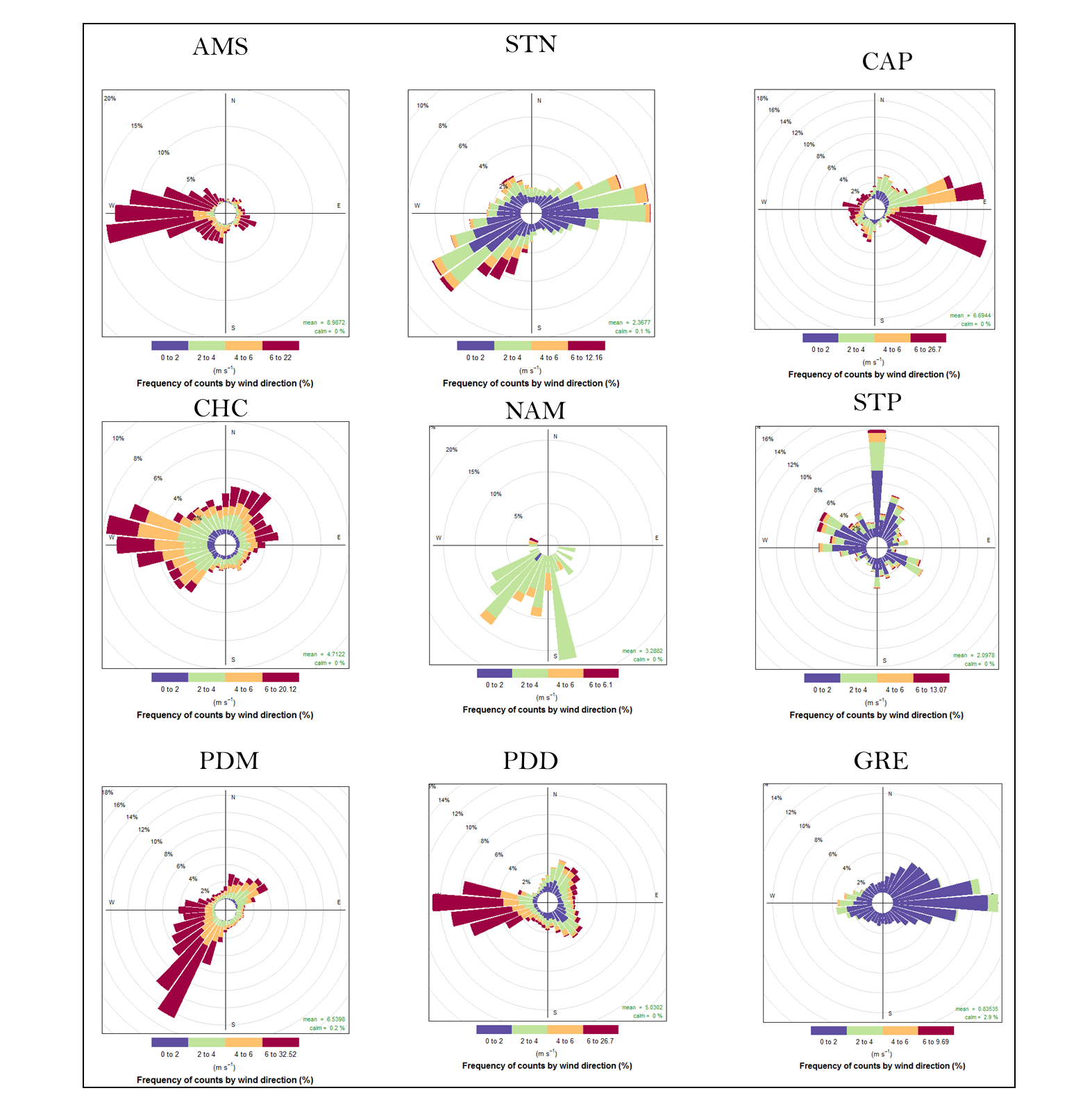


Supplementary Figure S5. Wind roses covering the sampling time at each site.


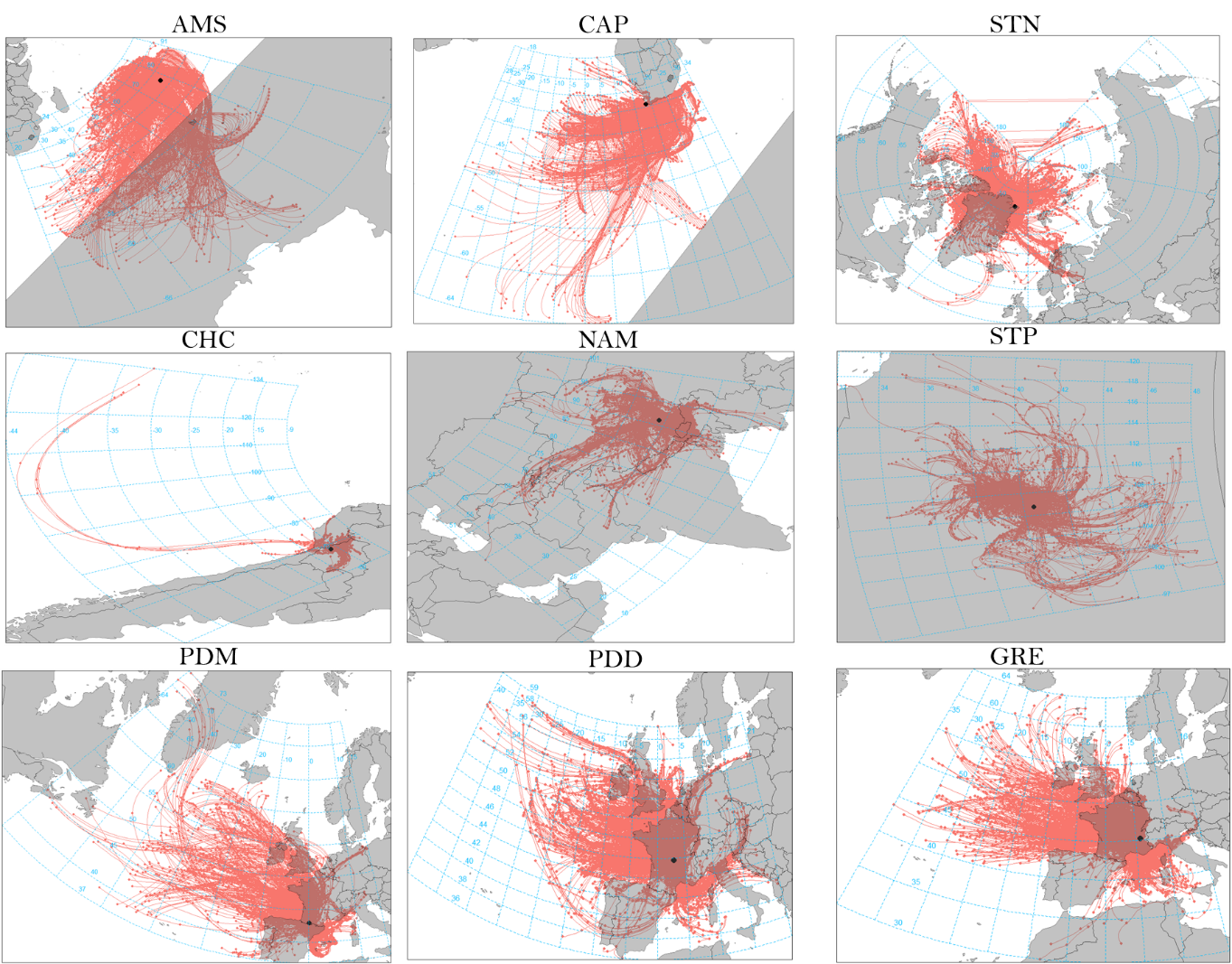


Supplementary Figure S6. Backward trajectories calculated over 3 days (maximum height from sea level: 1 km) at each site using HYSPLIT.


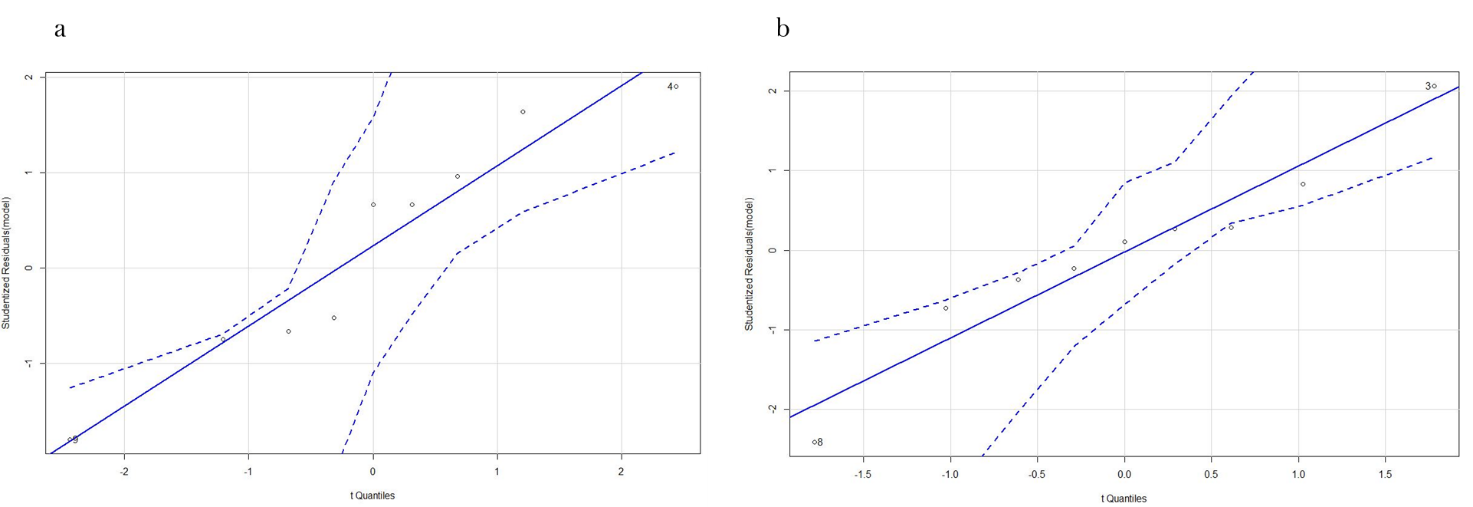


Supplementary Figure S7. Q-Q plots of the multiple linear regressions. (a) Comparison between the observed temporal variability values based on bacterial communities and the theoretical model (explaining variables: wind direction variability between and within weeks, temperature variability between weeks and landscape evenness); (b) Comparison between the observed temporal variability values based on fungal communities and the theoretical model (explaining variables: maximum wind speed and temperature variability between weeks).

**Supplementary Information Tables**

Supplementary Table S1. Sampling starting date, collected volume, raw read number and percentage of annotated sequences using RDP Calssifier for both *16s rRNA* gene and ITS sequencings for each sample. Blank cells indicate that the sample was removed from the dataset because of low read number (<6000 reads, except arctic Station-Nord samples which might have <6000 reads).

|  |  |  |  | ***16s rRNA* gene sequencing** | | **ITS sequencing** | |
| --- | --- | --- | --- | --- | --- | --- | --- |
| **Sample name** | **Site number** | **Standardized collected air volume (m^3^)** | **Sampling starting date (ending date 7 days after, same hour) (month/day/year)** | **Raw read number** | **Percentage of filtered sequences annotated to the genus level using RDP Classifier (%)** | **Raw read number** | **Percentage of filtered sequences annotated to the species level using RDP Classifier (%)** |
| AMS_10/09/2016 | AMS S1 | 5232 | 09/10/16 | 62953 | 61.5 | 36823 | 41.8 |
| AMS_17/09/2016 | AMS S2 | 5266 | 09/17/16 | 12741 | 58.9 | 37244 | 42.7 |
| AMS_24/09/2016 | AMS S3 | 4985 | 09/24/16 | 78891 | 50.7 | 6534 | 40.8 |
| AMS_01/10/2016 | AMS S4 | 5182 | 10/01/16 | 45843 | 46.9 | 21370 | 40.7 |
| AMS_08/10/2016 | AMS S5 | 4449 | 10/08/16 | 85337 | 51.5 | 31399 | 33.1 |
| AMS_14/10/2016 | AMS S6 | 5282 | 10/14/16 | 58765 | 49.2 | 29509 | 43.6 |
| AMS_21/10/2016 | AMS S7 | 5059 | 10/21/16 |  |  | 31671 | 41.5 |
| AMS_28/10/2016 | AMS S8 | 5153 | 10/28/16 | 83908 | 43.4 | 26055 | 42.1 |
| AMS_04/11/2016 | AMS S9 | 5035 | 11/04/16 | 26816 | 54.7 | 20906 | 42.8 |
| CAP_14/10/2016 | CAP S1 | 4630 | 10/14/16 | 29049 | 64.6 | 14092 | 55.3 |
| CAP_21/10/2016 | CAP S2 | 4679 | 10/21/16 | 126203 | 54.9 | 36185 | 49.7 |
| CAP_28/10/2016 | CAP S3 | 545 | 10/28/16 | 109434 | 57.8 | 32413 | 50.5 |
| CAP_04/11/2016 | CAP S4 | 4695 | 11/04/16 | 150604 | 55.7 | 35135 | 58.2 |
| CAP_11/11/2016 | CAP S5 | 4667 | 11/11/16 | 108178 | 63.0 | 34074 | 47.4 |
| CAP_18/11/2016 | CAP S6 | 4673 | 11/18/16 | 108286 | 61.3 | 20212 | 47.8 |
| CAP_25/11/2016 | CAP S7 | 4668 | 11/25/16 | 129262 | 58.1 | 22404 | 41.1 |
| CHC_01/07/2016 | CHC S1 | 1284 | 07/01/16 | 90867 | 57.5 | 22514 | 32.4 |
| CHC_09/09/2017 | CHC S10 | 1157 | 09/09/17 | 192437 | 62.4 | 24981 | 31.8 |
| CHC_16/09/2016 | CHC S11 | 824 | 09/16/16 | 8310 | 64.8 |  |  |
| CHC_23/09/2016 | CHC S12 | 1148 | 09/23/16 | 22575 | 63.3 | 12635 | 24.0 |
| CHC_30/09/2016 | CHC S13 | 1163 | 09/30/16 | 189657 | 58.4 | 59249 | 27.2 |
| CHC_14/10/2016 | CHC S14 | 1151 | 10/14/16 | 38877 | 64.1 |  |  |
| CHC_21/10/2016 | CHC S15 | 1154 | 10/21/16 | 90828 |  | 35825 | 23.8 |
| CHC_28/10/2016 | CHC S16 | 1220 | 10/28/16 | 14370 |  | 38071 | 24.5 |
| CHC_08/07/2016 | CHC S2 | 1284 | 07/08/16 | 180240 | 60.2 |  |  |
| CHC_15/07/2016 | CHC S3 | 1289 | 07/15/16 | 89332 | 61.2 | 27833 | 30.5 |
| CHC_29/07/2016 | CHC S4 | 1155 | 07/29/16 | 25325 | 58.6 |  |  |
| CHC_05/08/2016 | CHC S5 | 1157 | 08/05/16 | 90048 | 61.0 | 24885 | 32.3 |
| CHC_12/08/2016 | CHC S6 | 1160 | 08/12/16 | 163307 | 60.8 | 26773 | 28.7 |
| CHC_19/08/2016 | CHC S7 | 1158 | 08/19/16 | 94984 | 60.4 | 32254 | 29.2 |
| CHC_26/08/2016 | CHC S8 | 1158 | 08/26/16 | 112875 | 59.1 | 40067 | 32.5 |
| CHC_02/09/2016 | CHC S9 | 1156 | 09/02/16 | 48009 | 56.9 | 32250 | 30.0 |
| GRE_03/07/2017 | GRE S16 | 4688 | 07/03/17 | 42508 | 62.5 | 37691 | 38.6 |
| GRE_10/07/2017 | GRE S17 | 4717 | 07/10/17 | 50677 | 63.7 | 40657 | 24.8 |
| GRE_17/07/2017 | GRE S18 | 4677 | 07/17/17 | 64385 | 61.7 | 35547 | 34.4 |
| GRE_24/07/2017 | GRE S19 | 4718 | 07/24/17 | 34806 | 60.9 | 20997 | 52.5 |
| GRE_31/07/2017 | GRE S20 | 4665 | 07/31/17 | 53090 | 68.2 | 27444 | 49.3 |
| GRE_07/08/2017 | GRE S21 | 4762 | 08/07/17 | 37646 | 60.4 | 27978 | 48.2 |
| GRE_14/08/2017 | GRE S22 | 4729 | 08/14/17 | 57103 | 64.5 | 30992 | 42.8 |
| GRE_21/08/2017 | GRE S23 | 4707 | 08/21/17 | 35248 | 65.3 | 19205 | 54.0 |
| GRE_28/08/2017 | GRE S24 | 4744 | 08/28/17 | 49012 | 62.4 | 37441 | 51.4 |
| GRE_04/09/2017 | GRE S25 | 4742 | 09/04/17 | 31545 | 61.8 | 34026 | 52.4 |
| NAM_17/05/2017 | NAM S1 | 5511 | 05/17/17 | 44324 | 50.3 | 16320 | 34.4 |
| NAM_25/05/2017 | NAM S2 | 5503 | 05/25/17 | 41233 | 54.9 | 19277 | 32.7 |
| NAM_02/06/2017 | NAM S3 | 5513 | 06/02/17 | 50773 | 50.2 | 21024 | 29.1 |
| NAM_13/06/2017 | NAM S4 | 4218 | 06/13/17 | 47395 | 51.1 | 23504 | 31.8 |
| NAM_20/06/2017 | NAM S5 | 5418 | 06/20/17 | 19614 | 57.4 | 19270 | 34.0 |
| NAM_29/06/2017 | NAM S6 | 5415 | 06/29/17 |  |  | 20615 | 23.8 |
| NAM_07/07/2017 | NAM S7 | 5483 | 07/07/17 | 42211 | 57.2 | 21025 | 19.7 |
| NAM_14/07/2017 | NAM S8 | 5413 | 07/14/17 | 52028 | 58.5 | 12525 | 23.5 |
| NAM_21/07/2017 | NAM S9 | 5465 | 07/21/17 | 48018 | 55.1 | 22636 | 33.6 |
| PDD_29/06/2016 | PDD S1 | 8610 | 06/29/16 | 63115 | 67.9 | 15813 | 48.5 |
| PDD_01/09/2016 | PDD S10 | 8610 | 09/01/16 | 42068 | 60.8 | 25941 | 17.8 |
| PDD_07/09/2016 | PDD S11 | 8578 | 09/07/16 | 45669 | 53.3 | 39370 | 15.7 |
| PDD_14/09/2016 | PDD S12 | 8675 | 09/14/16 | 59341 | 56.7 | 26614 | 46.3 |
| PDD_21/09/2016 | PDD S13 | 8463 | 09/21/16 | 58601 | 60.8 | 42164 | 12.3 |
| PDD_06/07/2016 | PDD S2 | 8230 | 07/06/16 | 51661 | 42.2 | 17016 | 44.1 |
| PDD_13/07/2016 | PDD S3 | 8591 | 07/13/16 | 51967 | 61.9 | 27368 | 38.8 |
| PDD_20/07/2016 | PDD S4 | 8769 | 07/20/16 | 82471 | 65.7 | 31202 | 54.7 |
| PDD_03/08/2016 | PDD S6 | 8447 | 08/03/16 | 41648 | 67.1 | 21891 | 24.3 |
| PDD_10/08/2016 | PDD S7 | 8476 | 08/10/16 | 50687 | 65.3 | 25038 | 24.4 |
| PDD_17/08/2016 | PDD S8 | 8552 | 08/17/16 | 39687 | 64.3 | 24683 | 20.0 |
| PDD_24/08/2016 | PDD S9 | 8817 | 08/24/16 | 57870 | 66.3 | 20751 | 18.5 |
| PDM_20/06/2016 | PDM S1 | 9664 | 06/20/16 | 51437 | 82.0 | 16290 | 30.3 |
| PDM_23/08/2016 | PDM S10 | 7956 | 08/23/16 | 54036 | 61.2 | 27868 | 18.1 |
| PDM_13/09/2016 | PDM S11 | 7931 | 09/13/16 | 85531 | 65.1 | 26033 | 17.0 |
| PDM_20/09/2016 | PDM S12 | 7853 | 09/20/16 | 28412 | 62.5 | 34125 | 11.7 |
| PDM_06/09/2016 | PDM S13 | 7867 | 09/06/16 | 66574 | 61.6 | 20450 | 15.0 |
| PDM_27/09/2641 | PDM S14 | 7985 | 09/27/41 | 29506 | 60.7 | 21092 | 13.9 |
| PDM_29/06/2016 | PDM S2 | 6803 | 06/29/16 | 22167 | 70.0 |  |  |
| PDM_12/07/2016 | PDM S4 | 7550 | 07/12/16 | 171749 | 72.0 | 27356 | 18.5 |
| PDM_19/07/2016 | PDM S5 | 8040 | 07/19/16 | 38153 | 64.2 | 13360 | 32.2 |
| PDM_26/07/2016 | PDM S6 | 7794 | 07/26/16 | 41907 | 63.9 | 29215 | 18.4 |
| PDM_02/08/2016 | PDM S7 | 8103 | 08/02/16 | 61278 | 61.4 | 9943 | 19.2 |
| PDM_09/08/2016 | PDM S8 | 7747 | 09/08/16 |  |  | 8295 | 25.0 |
| PDM_16/08/2016 | PDM S9 | 8100 | 08/16/16 | 54431 | 67.1 | 4386 | 25.9 |
| STN_27/03/2017 | STN S1 | 5153 | 03/27/17 | 8937 | 70.7 |  |  |
| STN_29/05/2017 | STN S10 | 5273 | 05/29/17 | 155 | 20.6 | 2638 | 43.2 |
| STN_05/06/2017 | STN S11 | 5333 | 06/05/17 | 10926 | 67.9 | 3166 | 25.5 |
| STN_12/06/2017 | STN S12 | 5319 | 06/12/17 | 15440 | 63.7 | 25940 | 29.1 |
| STN_19/06/2017 | STN S13 | 5315 | 06/19/17 | 28385 | 61.6 | 17579 | 30.8 |
| STN_03/04/2017 | STN S2 | 5130 | 04/03/17 | 970 | 24.5 |  |  |
| STN_10/04/2017 | STN S3 | 5085 | 04/10/17 | 8738 | 66.7 |  |  |
| STN_17/04/2017 | STN S4 | 5128 | 04/17/17 | 1315 | 58.6 |  |  |
| STN_24/04/2017 | STN S5 | 5171 | 04/24/17 | 5856 | 64.4 |  |  |
| STN_01/05/2016 | STN S6 | 5154 | 05/01/16 | 2343 | 64.7 |  |  |
| STN_08/05/2017 | STN S7 | 5186 | 05/08/17 | 1744 | 74.1 |  |  |
| STN_15/05/2017 | STN S8 | 5246 | 05/15/17 | 47026 | 43.1 |  |  |
| STN_22/05/2017 | STN S9 | 5257 | 05/22/17 | 1951 | 64.5 |  |  |
| STP_14/07/2017 | STP S1 | 11213 | 07/14/17 | 38363 | 64.0 | 17061 | 17.6 |
| STP_21/07/2017 | STP S2 | 9333 | 07/21/17 | 36037 | 67.0 | 23616 | 13.5 |
| STP_28/07/2017 | STP S3 | 5702 | 07/28/17 | 43072 | 63.3 | 30811 | 18.4 |
| STP_04/08/2017 | STP_S4 | 5702 | 04/08/17 |  |  | 13396 | 30.9 |
| STP_11/08/2017 | STP_S5 | 5702 | 11/08/17 |  |  | 15977 | 24.8 |
| STP_18/08/2017 | STP_S6 | 5702 | 08/18/17 |  |  | 26082 | 29.5 |
| STP_25/08/2017 | STP S7 | 5702 | 08/25/17 | 27320 | 62.2 | 33239 | 34.0 |

Supplementary Table S2. Presentation of the different MODIS land covers from *Friedl et al., 2002*^6^.


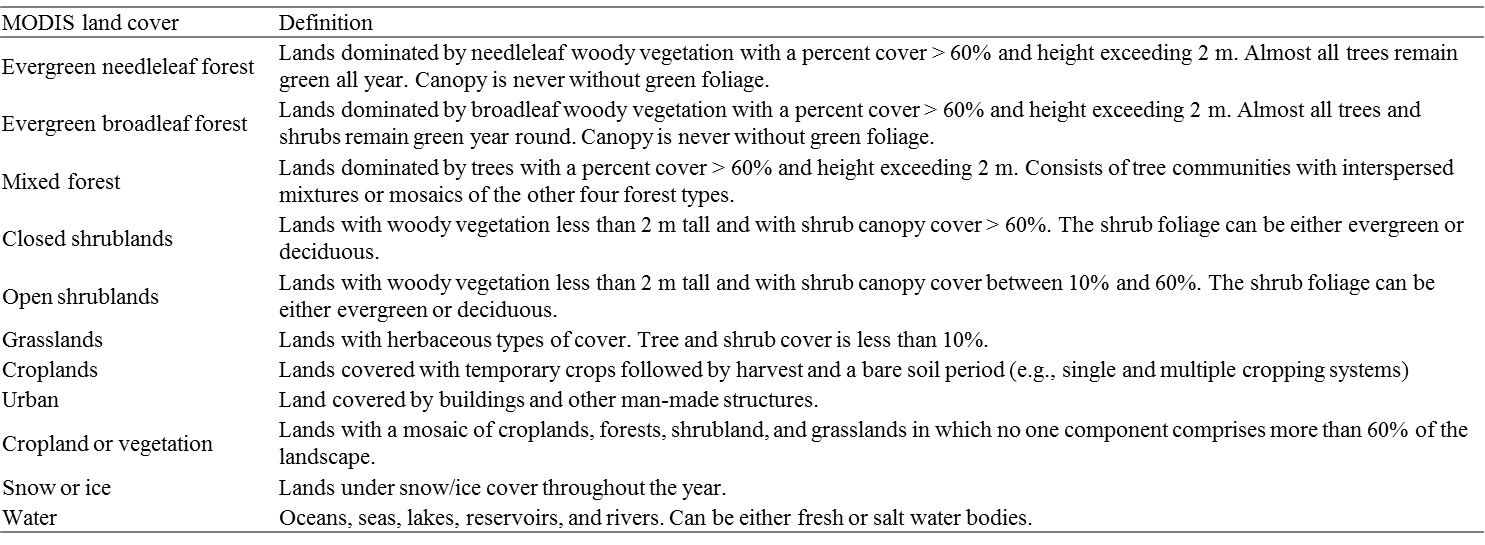


Supplementary Table S3. Estimation of mean bacterial cell concentration per cubic meter of air in near-surface air above the different landscapes reported in *Burrows et al., 2009*^5,7^. The « best estimates » have been used.


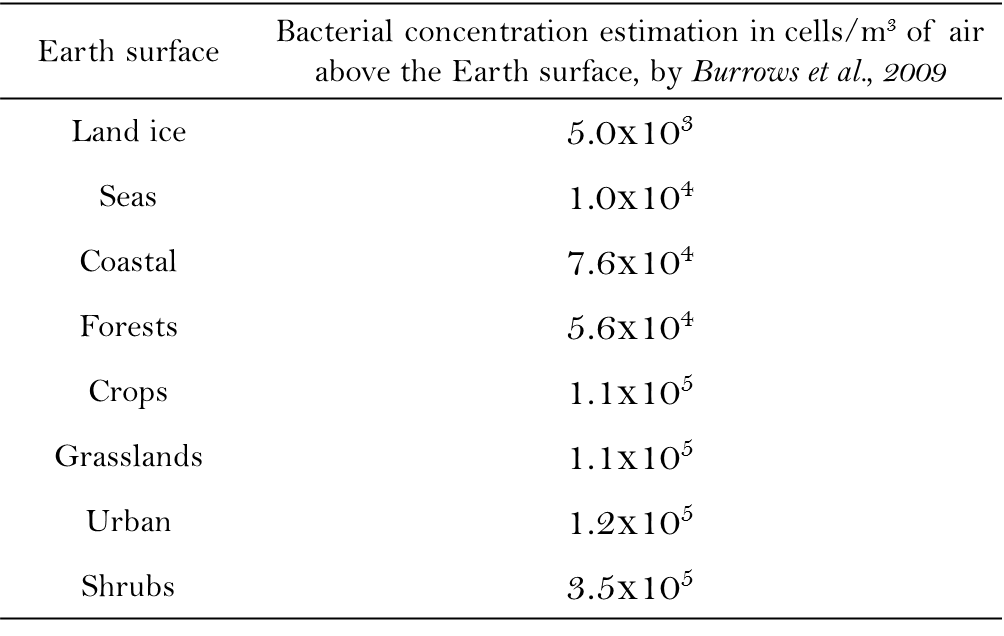


Supplementary Table S4. Total abundance (number of annotated sequences), contribution in each site and relative abundance per site of the first fifty most abundant bacterial genera

|  |  |  |  | Genus contribution in each site (%) | | | | | | | | | | | Relative abundance of each genus per site (%) | | | | | | | | | | | |
| --- | --- | --- | --- | --- | --- | --- | --- | --- | --- | --- | --- | --- | --- | --- | --- | --- | --- | --- | --- | --- | --- | --- | --- | --- | --- | --- |
| Bacterial genus | Bacterial family | Total number of each genus | Genus contribution (%) | AMS_% | | CAP_% | CHC_% | GRE_% | NAM_% | PDD_% | PDM_% | | STN_% | STP_% | AMS | CAP | | CHC | GRE | | NAM | | PDD | PDM | STN | STP |
| Total number of sequences per site |  |  |  |  |  | |  |  |  |  | |  |  |  | 220769 | | 419353 | 810507 | | 232126 | 172895 | 1107323 | | 240389 | 72244 | 52500 |
| Bacillus | Bacillaceae | 273852 | 8.23 | 0.03 | 11.67 | | 13.63 | 4.07 | 0.71 | 63.21 | | 5.44 | 0.15 | 1.08 | 0.03 | 7.62 | | 4.61 | | 4.8 | 1.12 | 15.63 | | 6.2 | 0.57 | 5.63 |
| Sphingomonas | Sphingomonadaceae | 187036 | 5.62 | 6.83 | 7.86 | | 9.35 | 15.13 | 3.75 | 43.7 | | 9.69 | 2.12 | 1.57 | 5.78 | 3.5 | | 2.16 | | 12.19 | 4.06 | 7.38 | | 7.54 | 5.48 | 5.58 |
| Hymenobacter | Cytophagaceae | 143844 | 4.32 | 3.94 | 2.29 | | 8.78 | 4.65 | 2.83 | 70.7 | | 3.73 | 2.42 | 0.66 | 2.56 | 0.79 | | 1.56 | | 2.88 | 2.35 | 9.18 | | 2.23 | 4.82 | 1.8 |
| Romboutsia | Peptostreptococcaceae | 92185 | 2.77 | 0.03 | 3.37 | | 30.62 | 3.24 | 1.53 | 53.89 | | 6.3 | 0.25 | 0.77 | 0.01 | 0.74 | | 3.48 | | 1.29 | 0.81 | 4.49 | | 2.41 | 0.32 | 1.36 |
| Methylobacterium | Methylobacteriaceae | 87488 | 2.63 | 2.1 | 2.99 | | 9.81 | 10.16 | 0.48 | 64.25 | | 5.57 | 3.91 | 0.73 | 0.83 | 0.62 | | 1.06 | | 3.83 | 0.24 | 5.08 | | 2.03 | 4.74 | 1.22 |
| Clostridium_sensu_stricto | Clostridiaceae | 72660 | 2.18 | 0.07 | 17.05 | | 17.46 | 3.78 | 0.32 | 46.81 | | 13.08 | 0.65 | 0.8 | 0.02 | 2.95 | | 1.56 | | 1.18 | 0.13 | 3.07 | | 3.95 | 0.65 | 1.1 |
| Thermoactinomyces | Thermoactinomycetaceae | 62956 | 1.89 | 0.1 | 0.9 | | 1.93 | 14.45 | 0.06 | 80.94 | | 1.33 | 0.07 | 0.21 | 0.03 | 0.13 | | 0.15 | | 3.92 | 0.02 | 4.6 | | 0.35 | 0.06 | 0.25 |
| Segetibacter | Chitinophagaceae | 50313 | 1.51 | 0.03 | 0.85 | | 80.4 | 0.33 | 8.41 | 3.27 | | 3.45 | 0.56 | 2.7 | 0.01 | 0.1 | | 4.99 | | 0.07 | 2.45 | 0.15 | | 0.72 | 0.39 | 2.58 |
| Turicibacter | Erysipelotrichaceae | 43272 | 1.3 | 0.02 | 5.08 | | 27.17 | 3.12 | 0.67 | 50.52 | | 11 | 0.26 | 2.16 | 0 | 0.52 | | 1.45 | | 0.58 | 0.17 | 1.97 | | 1.98 | 0.15 | 1.78 |
| Modestobacter | Geodermatophilaceae | 40533 | 1.22 | 0.01 | 1.1 | | 71.28 | 1.42 | 19.63 | 1.69 | | 2.45 | 0.41 | 2 | 0 | 0.11 | | 3.56 | | 0.25 | 4.6 | 0.06 | | 0.41 | 0.23 | 1.55 |
| Clostridium_XI | Peptostreptococcaceae | 39826 | 1.2 | 0.32 | 45.31 | | 12.33 | 0.93 | 0.41 | 37.96 | | 2.15 | 0.01 | 0.56 | 0.06 | 4.3 | | 0.61 | | 0.16 | 0.1 | 1.37 | | 0.36 | 0.01 | 0.43 |
| Blastococcus | Geodermatophilaceae | 37783 | 1.14 | 0.17 | 5.16 | | 45.98 | 5.17 | 21.08 | 5.08 | | 12.27 | 2.25 | 2.83 | 0.03 | 0.46 | | 2.14 | | 0.84 | 4.61 | 0.17 | | 1.93 | 1.18 | 2.04 |
| Sporosarcina | Planococcaceae | 36476 | 1.1 | 0.18 | 18.53 | | 12.13 | 2.06 | 1.44 | 58.62 | | 5.46 | 0.61 | 0.96 | 0.03 | 1.61 | | 0.55 | | 0.32 | 0.3 | 1.93 | | 0.83 | 0.31 | 0.67 |
| Gemmatimonas | Gemmatimonadaceae | 33981 | 1.02 | 0.05 | 10.09 | | 41.84 | 2.81 | 15.8 | 7.9 | | 15.01 | 3.86 | 2.66 | 0.01 | 0.82 | | 1.75 | | 0.41 | 3.1 | 0.24 | | 2.12 | 1.81 | 1.72 |
| Nocardioides | Nocardioidaceae | 32978 | 0.99 | 3.13 | 12.97 | | 35.96 | 8.92 | 6.33 | 15.03 | | 10.29 | 4.87 | 2.49 | 0.47 | 1.02 | | 1.46 | | 1.27 | 1.21 | 0.45 | | 1.41 | 2.22 | 1.57 |
| Arthrobacter | Micrococcaceae | 31139 | 0.94 | 0.2 | 6.29 | | 55.43 | 3.16 | 6.56 | 11.33 | | 12 | 1.09 | 3.94 | 0.03 | 0.47 | | 2.13 | | 0.42 | 1.18 | 0.32 | | 1.55 | 0.47 | 2.34 |
| Lysinibacillus | Planococcaceae | 26959 | 0.81 | 0 | 7.12 | | 10.75 | 7.22 | 0.76 | 65.45 | | 7.16 | 0.02 | 1.51 | 0 | 0.46 | | 0.36 | | 0.84 | 0.12 | 1.59 | | 0.8 | 0.01 | 0.78 |
| Pseudomonas | Pseudomonadaceae | 26068 | 0.78 | 2.06 | 6.5 | | 12.08 | 10.96 | 0.85 | 61.14 | | 4.93 | 0.33 | 1.17 | 0.24 | 0.4 | | 0.39 | | 1.23 | 0.13 | 1.44 | | 0.53 | 0.12 | 0.58 |
| Massilia | Oxalobacteraceae | 25505 | 0.77 | 0.33 | 3.68 | | 22.55 | 17.93 | 2.47 | 34.12 | | 13 | 1.76 | 4.14 | 0.04 | 0.22 | | 0.71 | | 1.97 | 0.36 | 0.79 | | 1.38 | 0.62 | 2.01 |
| Rubellimicrobium | Rhodobacteraceae | 25361 | 0.76 | 0.02 | 9 | | 40.2 | 13.53 | 14.77 | 8.98 | | 9.21 | 2.1 | 2.19 | 0 | 0.54 | | 1.26 | | 1.48 | 2.17 | 0.21 | | 0.97 | 0.74 | 1.06 |
| Corynebacterium | Corynebacteriaceae | 25258 | 0.76 | 0.26 | 8.07 | | 57.17 | 7.38 | 6.24 | 9.16 | | 5.85 | 3.42 | 2.46 | 0.03 | 0.49 | | 1.78 | | 0.8 | 0.91 | 0.21 | | 0.61 | 1.19 | 1.18 |
| Kallotenue | Kallotenuaceae | 24017 | 0.72 | 0.01 | 1.53 | | 82.41 | 1.1 | 6.87 | 1.74 | | 4.8 | 0.1 | 1.44 | 0 | 0.09 | | 2.44 | | 0.11 | 0.95 | 0.04 | | 0.48 | 0.03 | 0.66 |
| GpI | Family I | 22539 | 0.68 | 0.15 | 6.15 | | 34.33 | 6.57 | 21.62 | 24.5 | | 1.4 | 4.32 | 0.97 | 0.01 | 0.33 | | 0.95 | | 0.64 | 2.82 | 0.5 | | 0.13 | 1.35 | 0.42 |
| Domibacillus | Bacillaceae | 21850 | 0.66 | 0.01 | 5.63 | | 6.76 | 1.37 | 0.34 | 72.77 | | 11.64 | 0.65 | 0.82 | 0 | 0.29 | | 0.18 | | 0.13 | 0.04 | 1.44 | | 1.06 | 0.2 | 0.34 |
| Pedobacter | Sphingobacteriaceae | 21674 | 0.65 | 2.69 | 2.54 | | 13.87 | 12.28 | 1.73 | 56.59 | | 7.01 | 1.29 | 1.99 | 0.26 | 0.13 | | 0.37 | | 1.15 | 0.22 | 1.11 | | 0.63 | 0.39 | 0.82 |
| Aquisphaera | Planctomycetaceae | 21573 | 0.65 | 0.68 | 4.58 | | 61.16 | 2.48 | 11.38 | 5.99 | | 5.81 | 5.22 | 2.7 | 0.07 | 0.24 | | 1.63 | | 0.23 | 1.42 | 0.12 | | 0.52 | 1.56 | 1.11 |
| Gp6 | - | 20902 | 0.63 | 0.12 | 13 | | 27.06 | 6.07 | 14.4 | 13.34 | | 17.18 | 6.06 | 2.77 | 0.01 | 0.65 | | 0.7 | | 0.55 | 1.74 | 0.25 | | 1.49 | 1.75 | 1.1 |
| Gp16 | - | 20886 | 0.63 | 0.08 | 6.45 | | 36.46 | 4.12 | 15.01 | 12.97 | | 16.48 | 6.64 | 1.79 | 0.01 | 0.32 | | 0.94 | | 0.37 | 1.81 | 0.24 | | 1.43 | 1.92 | 0.71 |
| Marmoricola | Nocardioidaceae | 20014 | 0.6 | 2.21 | 11.98 | | 44.44 | 6.86 | 8.87 | 12.45 | | 8.27 | 2.58 | 2.33 | 0.2 | 0.57 | | 1.1 | | 0.59 | 1.03 | 0.22 | | 0.69 | 0.72 | 0.89 |
| Tumebacillus | Alicyclobacillaceae | 19727 | 0.59 | 0.01 | 6.34 | | 5.01 | 3.53 | 0.2 | 78.26 | | 5.51 | 0.03 | 1.12 | 0 | 0.3 | | 0.12 | | 0.3 | 0.02 | 1.39 | | 0.45 | 0.01 | 0.42 |
| Anaerobacter | Ruminococcaceae | 19298 | 0.58 | 0.12 | 15.49 | | 16.58 | 3.39 | 0.4 | 48.8 | | 14.44 | 0.09 | 0.69 | 0.01 | 0.71 | | 0.39 | | 0.28 | 0.05 | 0.85 | | 1.16 | 0.02 | 0.25 |
| Nakamurella | Nakamurellaceae | 18866 | 0.57 | 0.61 | 1.41 | | 16.44 | 2.33 | 0.82 | 74.03 | | 3.15 | 0.22 | 0.99 | 0.05 | 0.06 | | 0.38 | | 0.19 | 0.09 | 1.26 | | 0.25 | 0.06 | 0.36 |
| Geodermatophilus | Geodermatophilaceae | 18516 | 0.56 | 0.75 | 5.28 | | 21.72 | 5.47 | 27.64 | 14.23 | | 15.23 | 2.93 | 6.77 | 0.06 | 0.23 | | 0.5 | | 0.44 | 2.96 | 0.24 | | 1.17 | 0.75 | 2.39 |
| Planomicrobium | Planococcaceae | 18505 | 0.56 | 0 | 1.1 | | 72.73 | 1.6 | 7.71 | 3.27 | | 9.53 | 0.02 | 4.03 | 0 | 0.05 | | 1.66 | | 0.13 | 0.83 | 0.05 | | 0.73 | 0.01 | 1.42 |
| Terrisporobacter | Peptostreptococcaceae | 18212 | 0.55 | 0.02 | 8.15 | | 23.01 | 2.6 | 0.43 | 48.91 | | 16.62 | 0.01 | 0.24 | 0 | 0.35 | | 0.52 | | 0.2 | 0.05 | 0.8 | | 1.26 | 0 | 0.08 |
| Kocuria | Micrococcaceae | 17994 | 0.54 | 0.04 | 6.2 | | 77.68 | 3.01 | 1.91 | 2.36 | | 4.83 | 0.31 | 3.66 | 0 | 0.27 | | 1.72 | | 0.23 | 0.2 | 0.04 | | 0.36 | 0.08 | 1.25 |
| Paenibacillus | Paenibacillaceae | 17951 | 0.54 | 0.03 | 9.43 | | 13.98 | 4.95 | 0.53 | 65.33 | | 4.92 | 0.03 | 0.8 | 0 | 0.4 | | 0.31 | | 0.38 | 0.06 | 1.06 | | 0.37 | 0.01 | 0.27 |
| Streptomyces | Streptomycetaceae | 17828 | 0.54 | 0.83 | 61.18 | | 10.07 | 16.1 | 2.05 | 4.52 | | 4.15 | 0.09 | 1.01 | 0.07 | 2.6 | | 0.22 | | 1.24 | 0.21 | 0.07 | | 0.31 | 0.02 | 0.34 |
| Geminicoccus | - | 17678 | 0.53 | 0.53 | 5.71 | | 23.01 | 2.73 | 56.46 | 2.33 | | 6.68 | 1.38 | 1.18 | 0.04 | 0.24 | | 0.5 | | 0.21 | 5.77 | 0.04 | | 0.49 | 0.34 | 0.4 |
| Roseomonas | Acetobacteraceae | 17545 | 0.53 | 0.56 | 7.89 | | 38.38 | 7.31 | 9.69 | 14.48 | | 7.19 | 11.38 | 3.12 | 0.04 | 0.33 | | 0.83 | | 0.55 | 0.98 | 0.23 | | 0.52 | 2.76 | 1.04 |
| Microvirga | Methylobacteriaceae | 17530 | 0.53 | 0 | 6.09 | | 30.29 | 3.57 | 35.22 | 7.42 | | 14.5 | 0.22 | 2.69 | 0 | 0.25 | | 0.66 | | 0.27 | 3.57 | 0.12 | | 1.06 | 0.05 | 0.9 |
| Gaiella | Rubrobacteraceae | 17391 | 0.52 | 0.1 | 5.52 | | 21.48 | 6.21 | 17 | 17.91 | | 24.18 | 6.12 | 1.49 | 0.01 | 0.23 | | 0.46 | | 0.47 | 1.71 | 0.28 | | 1.75 | 1.47 | 0.49 |
| Rubritalea | Rubritaleaceae | 16949 | 0.51 | 49.96 | 49.85 | | 0.01 | 0.03 | 0 | 0.11 | | 0 | 0 | 0.05 | 3.84 | 2.01 | | 0 | | 0 | 0 | 0 | | 0 | 0 | 0.02 |
| Sphingobacterium | Sphingobacteriaceae | 16777 | 0.5 | 0.01 | 91.94 | | 1.17 | 2.65 | 0.46 | 2.66 | | 0.9 | 0 | 0.21 | 0 | 3.68 | | 0.02 | | 0.19 | 0.05 | 0.04 | | 0.06 | 0 | 0.07 |
| Skermanella | Rhodospirillaceae | 16319 | 0.49 | 0 | 2.87 | | 45.74 | 3.59 | 21.48 | 5.48 | | 14.07 | 4.81 | 1.95 | 0 | 0.11 | | 0.92 | | 0.25 | 2.03 | 0.08 | | 0.96 | 1.09 | 0.61 |
| Gp3 |  | 16292 | 0.49 | 0.06 | 6.3 | | 70.8 | 1.52 | 5.71 | 5.68 | | 6.21 | 2.12 | 1.61 | 0 | 0.24 | | 1.42 | | 0.11 | 0.54 | 0.08 | | 0.42 | 0.48 | 0.5 |
| Paracoccus | Rhodobacteraceae | 16202 | 0.49 | 2.17 | 22.55 | | 46.54 | 9.96 | 2.04 | 7.65 | | 6.79 | 1.08 | 1.22 | 0.16 | 0.87 | | 0.93 | | 0.7 | 0.19 | 0.11 | | 0.46 | 0.24 | 0.38 |
| Acidisphaera | Acetobacteraceae | 15887 | 0.48 | 0.22 | 0.36 | | 13.57 | 0.57 | 0.14 | 84.38 | | 0.6 | 0.09 | 0.06 | 0.02 | 0.01 | | 0.27 | | 0.04 | 0.01 | 1.21 | | 0.04 | 0.02 | 0.02 |
| Phenylobacterium | Caulobacteraceae | 15797 | 0.47 | 0.81 | 11.92 | | 22.74 | 3.02 | 1.27 | 35.85 | | 6.9 | 16.43 | 1.06 | 0.06 | 0.45 | | 0.44 | | 0.21 | 0.12 | 0.51 | | 0.45 | 3.59 | 0.32 |
| Ornithinimicrobium | Intrasporangiaceae | 15755 | 0.47 | 0.06 | 12.19 | | 66.07 | 2.93 | 3.2 | 3.81 | | 7.88 | 0.3 | 3.56 | 0 | 0.46 | | 1.28 | | 0.2 | 0.29 | 0.05 | | 0.52 | 0.07 | 1.07 |

Supplementary Table S5. Total abundance (number of annotated sequences), contribution in each site and relative abundance per site of the first fifty most abundant fungal species

|  |  |  |  | Genus contribution in each site (%) | | | | | | | | | | Relative abundance of each genus per site (%) | | | | | | | | |
| --- | --- | --- | --- | --- | --- | --- | --- | --- | --- | --- | --- | --- | --- | --- | --- | --- | --- | --- | --- | --- | --- | --- |
| Fungal species | Fungal family | Total number of each genus | Genus contribution (%) | AMS_% | CAP_% | CHC_% | GRE_% | NAM_% | PDD_% | PDM_% | STN_% | STP_% | | AMS | CAP | CHC | GRE | NAM | PDD | PDM | STN | STP |
| Total number of sequences per site |  |  |  |  |  |  |  |  |  |  |  | |  | 98965 | 97585 | 108560 | 135543 | 51672 | 507133 | 39325 | 14920 | 39006 |
| Pseudotaeniolina globosa | Capnodiales_Incertae sedis | 59485 | 5.44 | 1.25 | 0.7 | 0.22 | 0.02 | 0.01 | 97.77 | 0.04 | 0 | | 0 | 0.75 | 0.42 | 0.12 | 0.01 | 0.01 | 11.47 | 0.07 | 0 | 0 |
| Cladophialophora proteae | Herpotrichiellaceae | 40075 | 3.67 | 0 | 0 | 0 | 0 | 0 | 99.99 | 0 | 0 | | 0 | 0 | 0 | 0 | 0 | 0 | 7.9 | 0 | 0 | 0 |
| Ustilago bullata | Ustilaginaceae | 35197 | 3.22 | 0 | 0.19 | 16.23 | 0.03 | 0.05 | 14.11 | 3.48 | 0 | | 65.9 | 0 | 0.07 | 5.26 | 0.01 | 0.04 | 0.98 | 3.12 | 0 | 59.47 |
| Alternaria sp BMP_2012 | Pleosporaceae | 28415 | 2.6 | 0 | 16.66 | 0.01 | 4.58 | 0 | 71.68 | 7 | 0.05 | | 0 | 0 | 4.85 | 0 | 0.96 | 0 | 4.02 | 5.06 | 0.1 | 0 |
| Botryotinia fuckeliana | Sclerotiniaceae | 26986 | 2.47 | 3.6 | 2.87 | 5.94 | 10.96 | 1.16 | 48.21 | 8.56 | 17.27 | | 1.42 | 0.98 | 0.79 | 1.48 | 2.18 | 0.6 | 2.57 | 5.88 | 31.23 | 0.98 |
| Cladophialophora minutissima | Herpotrichiellaceae | 20181 | 1.85 | 0 | 0 | 0.11 | 0 | 0 | 99.86 | 0.02 | 0 | | 0 | 0 | 0 | 0.02 | 0 | 0 | 3.97 | 0.01 | 0 | 0 |
| Naevala minutissima | Dermateaceae | 18791 | 1.72 | 0.01 | 0 | 0.01 | 6.39 | 0.45 | 83.2 | 4.08 | 5.1 | | 0.76 | 0 | 0 | 0 | 0.89 | 0.16 | 3.08 | 1.95 | 6.43 | 0.36 |
| Erysiphe alphitoides | Erysiphaceae | 15581 | 1.43 | 0.01 | 0.01 | 0.03 | 0.06 | 0.03 | 99.22 | 0.63 | 0 | | 0.03 | 0 | 0 | 0 | 0.01 | 0.01 | 3.05 | 0.25 | 0 | 0.01 |
| Preussia minima | Sporormiaceae | 15218 | 1.39 | 0 | 0.09 | 1.89 | 1.11 | 0.04 | 96.15 | 0.63 | 0 | | 0.09 | 0 | 0.01 | 0.27 | 0.12 | 0.01 | 2.89 | 0.24 | 0 | 0.03 |
| Lophodermium agathidis | Rhytismataceae | 15133 | 1.38 | 92.59 | 0.27 | 0.11 | 0.53 | 0.5 | 1.08 | 4.51 | 0.01 | | 0.39 | 14.16 | 0.04 | 0.02 | 0.06 | 0.15 | 0.03 | 1.73 | 0.01 | 0.15 |
| Coriolopsis trogii | Polyporaceae | 14697 | 1.35 | 0 | 1.07 | 0.33 | 94.69 | 0.22 | 1.51 | 2.17 | 0 | | 0.01 | 0 | 0.16 | 0.04 | 10.27 | 0.06 | 0.04 | 0.81 | 0 | 0.01 |
| Sporidiobolus sp JPS_2007a | Sporidiobolaceae | 12897 | 1.18 | 7.09 | 75.21 | 0.04 | 3.25 | 0.01 | 12.88 | 1.25 | 0.01 | | 0.26 | 0.92 | 9.94 | 0 | 0.31 | 0 | 0.33 | 0.41 | 0.01 | 0.09 |
| Toxicocladosporium rubrigenum | Capnodiales_Incertae sedis | 12331 | 1.13 | 1.1 | 13.09 | 29.25 | 1.18 | 40.35 | 10.99 | 2.72 | 0.43 | | 0.89 | 0.14 | 1.65 | 3.32 | 0.11 | 9.63 | 0.27 | 0.85 | 0.36 | 0.28 |
| Ustilago hordei | Ustilaginaceae | 11219 | 1.03 | 0 | 9.61 | 59.06 | 0.01 | 10.46 | 11.28 | 1.05 | 0 | | 8.53 | 0 | 1.1 | 6.1 | 0 | 2.27 | 0.25 | 0.3 | 0 | 2.45 |
| Sarcinomyces petricola | Pezizomycotina_Incertae sedis | 10886 | 1 | 0.02 | 0.12 | 1.4 | 0.06 | 0.45 | 97.61 | 0.31 | 0 | | 0.03 | 0 | 0.01 | 0.14 | 0.01 | 0.09 | 2.1 | 0.09 | 0 | 0.01 |
| Leptosphaerulina chartarum | Didymellaceae | 10866 | 0.99 | 0.02 | 16.54 | 3.43 | 5.37 | 0 | 69.83 | 4.77 | 0 | | 0.05 | 0 | 1.84 | 0.34 | 0.43 | 0 | 1.5 | 1.32 | 0 | 0.01 |
| Cryptococcus victoriae | Tremellaceae | 10825 | 0.99 | 7.64 | 12.38 | 9.53 | 2.84 | 1.26 | 57.93 | 7.5 | 0 | | 0.92 | 0.84 | 1.37 | 0.95 | 0.23 | 0.26 | 1.24 | 2.06 | 0 | 0.26 |
| Microdochium phragmitis | Hyponectriaceae | 10701 | 0.98 | 95.65 | 0.19 | 0.61 | 0 | 0.02 | 2.51 | 0.98 | 0.04 | | 0 | 10.34 | 0.02 | 0.06 | 0 | 0 | 0.05 | 0.27 | 0.03 | 0 |
| Epicoccum sp JJP_2009a | Pleosporales_Incertae sedis | 10308 | 0.94 | 0.56 | 0.28 | 2.58 | 2.49 | 0.62 | 88.32 | 4.74 | 0.06 | | 0.34 | 0.06 | 0.03 | 0.25 | 0.19 | 0.12 | 1.8 | 1.24 | 0.04 | 0.09 |
| Eurotium amstelodami | Trichocomaceae | 9965 | 0.91 | 0 | 0.09 | 2.17 | 2.09 | 0.17 | 94.17 | 1.19 | 0 | | 0.12 | 0 | 0.01 | 0.2 | 0.15 | 0.03 | 1.85 | 0.3 | 0 | 0.03 |
| Cladosporium langeronii | Mycosphaerellaceae | 9854 | 0.9 | 0.58 | 70.13 | 11.8 | 9.38 | 0.73 | 6.32 | 0.9 | 0 | | 0.15 | 0.06 | 7.08 | 1.07 | 0.68 | 0.14 | 0.12 | 0.23 | 0 | 0.04 |
| Chalara hyalocuspica | Pezizomycotina_Incertae sedis | 9675 | 0.89 | 91.49 | 0.23 | 0.06 | 0.6 | 0.85 | 5.75 | 0.81 | 0.18 | | 0.04 | 8.94 | 0.02 | 0.01 | 0.04 | 0.16 | 0.11 | 0.2 | 0.11 | 0.01 |
| Aureobasidium pullulans var subglaciale | Dothioraceae | 9601 | 0.88 | 0.01 | 0.08 | 86.67 | 0.26 | 5.17 | 4.67 | 1.9 | 0 | | 1.25 | 0 | 0.01 | 7.66 | 0.02 | 0.96 | 0.09 | 0.46 | 0 | 0.31 |
| Preussia intermedia | Sporormiaceae | 9413 | 0.86 | 0 | 0 | 33.72 | 0 | 15.97 | 48.56 | 0.72 | 0.01 | | 1.02 | 0 | 0 | 2.92 | 0 | 2.91 | 0.9 | 0.17 | 0.01 | 0.25 |
| Tranzscheliella hypodytes | Ustilaginaceae | 9405 | 0.86 | 0 | 0 | 96.12 | 0.02 | 0 | 1.45 | 0.52 | 0 | | 1.89 | 0 | 0 | 8.33 | 0 | 0 | 0.03 | 0.12 | 0 | 0.46 |
| Sistotrema alboluteum | Sistotremataceae | 9255 | 0.85 | 82.83 | 0.43 | 0.02 | 2.09 | 0.31 | 13.31 | 0.81 | 0.01 | | 0.18 | 7.75 | 0.04 | 0 | 0.14 | 0.06 | 0.24 | 0.19 | 0.01 | 0.04 |
| Baeospora myosura | Marasmiaceae | 8790 | 0.8 | 0 | 0 | 0.14 | 0.22 | 0 | 99.28 | 0.36 | 0 | | 0 | 0 | 0 | 0.01 | 0.01 | 0 | 1.72 | 0.08 | 0 | 0 |
| Sistotremastrum guttuliferum | Hydnodontaceae | 8695 | 0.8 | 86.6 | 2.4 | 0 | 3.75 | 0.06 | 6.36 | 0.54 | 0.01 | | 0.28 | 7.61 | 0.21 | 0 | 0.24 | 0.01 | 0.11 | 0.12 | 0.01 | 0.06 |
| Tremella foliacea | Tremellales_Incertae sedis | 8602 | 0.79 | 0.02 | 0 | 0.17 | 0.07 | 0 | 99.7 | 0.03 | 0 | | 0 | 0 | 0 | 0.01 | 0 | 0 | 1.69 | 0.01 | 0 | 0 |
| Aspergillus reptans | Trichocomaceae | 8571 | 0.78 | 0.01 | 0.04 | 9.61 | 1.58 | 0.92 | 87.06 | 0.64 | 0 | | 0.14 | 0 | 0 | 0.76 | 0.1 | 0.15 | 1.47 | 0.14 | 0 | 0.03 |
| Trichocladium asperum | Chaetomiaceae | 8265 | 0.76 | 0 | 0.19 | 15.33 | 0.22 | 38.73 | 44.49 | 0.92 | 0.01 | | 0.11 | 0 | 0.02 | 1.17 | 0.01 | 6.19 | 0.73 | 0.19 | 0.01 | 0.02 |
| Humicola nigrescens | Chaetomiaceae | 7754 | 0.71 | 0.03 | 0.08 | 1.99 | 0.05 | 59.8 | 35.79 | 0.43 | 1.65 | | 0.19 | 0 | 0.01 | 0.14 | 0 | 8.97 | 0.55 | 0.08 | 0.86 | 0.04 |
| Ganoderma applanatum | Ganodermataceae | 7677 | 0.7 | 0.01 | 0.01 | 0.01 | 60.79 | 0.39 | 10.52 | 11.84 | 0 | | 16.41 | 0 | 0 | 0 | 3.44 | 0.06 | 0.16 | 2.31 | 0 | 3.23 |
| Handkea utriformis | Lycoperdaceae | 7057 | 0.65 | 0 | 0 | 0 | 0.3 | 0.68 | 95.32 | 3.26 | 0 | | 0.44 | 0 | 0 | 0 | 0.02 | 0.09 | 1.33 | 0.58 | 0 | 0.08 |
| Aureobasidium pullulans | Dothioraceae | 7022 | 0.64 | 0.68 | 18.58 | 0.93 | 9.87 | 0 | 55.72 | 14 | 0 | | 0.21 | 0.05 | 1.34 | 0.06 | 0.51 | 0 | 0.77 | 2.5 | 0 | 0.04 |
| Phaeosphaeria herpotrichoides | Phaeosphaeriaceae | 6916 | 0.63 | 43.36 | 0.17 | 6.84 | 4.79 | 7.89 | 23.15 | 3.72 | 2.56 | | 7.52 | 3.03 | 0.01 | 0.44 | 0.24 | 1.06 | 0.32 | 0.65 | 1.19 | 1.33 |
| Physisporinus sanguinolentus | Meripilaceae | 6358 | 0.58 | 3.38 | 0.36 | 0.66 | 88.42 | 0.38 | 5.65 | 1.15 | 0 | | 0 | 0.22 | 0.02 | 0.04 | 4.15 | 0.05 | 0.07 | 0.19 | 0 | 0 |
| Ganoderma australe | Ganodermataceae | 6288 | 0.58 | 0.02 | 0 | 0.06 | 91.78 | 0 | 4.04 | 4.1 | 0 | | 0 | 0 | 0 | 0 | 4.26 | 0 | 0.05 | 0.66 | 0 | 0 |
| Lachnum ciliare | Hyaloscyphaceae | 6247 | 0.57 | 82.52 | 0.05 | 0 | 2.75 | 0 | 14.5 | 0.16 | 0.02 | | 0 | 5.21 | 0 | 0 | 0.13 | 0 | 0.18 | 0.03 | 0.01 | 0 |
| Psilolechia leprosa | Micareaceae | 5876 | 0.54 | 0 | 0 | 0.02 | 0 | 0 | 99.98 | 0 | 0 | | 0 | 0 | 0 | 0 | 0 | 0 | 1.16 | 0 | 0 | 0 |
| Blumeria graminis | Erysiphaceae | 5794 | 0.53 | 0 | 0.14 | 0.03 | 0.05 | 0 | 99.69 | 0.07 | 0 | | 0.02 | 0 | 0.01 | 0 | 0 | 0 | 1.14 | 0.01 | 0 | 0 |
| Schizophyllum commune | Schizophyllaceae | 5635 | 0.52 | 0.02 | 89.55 | 0.05 | 7.93 | 0 | 1.54 | 0.91 | 0 | | 0 | 0 | 5.17 | 0 | 0.33 | 0 | 0.02 | 0.13 | 0 | 0 |
| Pseudozyma shanxiensis | Ustilaginaceae | 5235 | 0.48 | 4.01 | 0.08 | 6.63 | 0.23 | 17.94 | 69.21 | 1.6 | 0 | | 0.31 | 0.21 | 0 | 0.32 | 0.01 | 1.82 | 0.71 | 0.21 | 0 | 0.04 |
| Microdochium majus | Hyponectriaceae | 5201 | 0.48 | 0 | 0.02 | 2.75 | 1.23 | 0.12 | 94.81 | 1.02 | 0 | | 0.06 | 0 | 0 | 0.13 | 0.05 | 0.01 | 0.97 | 0.13 | 0 | 0.01 |
| Pleospora alfalfae | Pleosporaceae | 5177 | 0.47 | 1.51 | 47.94 | 1.95 | 0.41 | 0 | 32.82 | 14.93 | 0 | | 0.44 | 0.08 | 2.54 | 0.09 | 0.02 | 0 | 0.34 | 1.97 | 0 | 0.06 |
| Podospora pleiospora | Lasiosphaeriaceae | 4938 | 0.45 | 0 | 0 | 4.35 | 0 | 0.02 | 95.38 | 0.22 | 0 | | 0.02 | 0 | 0 | 0.2 | 0 | 0 | 0.93 | 0.03 | 0 | 0 |
| Ypsilina graminea | Helotiaceae | 4785 | 0.44 | 95.01 | 0.02 | 0.04 | 0 | 0.02 | 4.79 | 0.1 | 0.02 | | 0 | 4.59 | 0 | 0 | 0 | 0 | 0.05 | 0.01 | 0.01 | 0 |
| Ulocladium chartarum | Pleosporaceae | 4750 | 0.43 | 0 | 0.08 | 84.91 | 0.06 | 8.08 | 2.27 | 3.28 | 0 | | 1.31 | 0 | 0 | 3.71 | 0 | 0.74 | 0.02 | 0.4 | 0 | 0.16 |
| Peniophora cinerea | Peniophoraceae | 4564 | 0.42 | 0.2 | 5 | 0.31 | 85.78 | 0 | 7.32 | 1.29 | 0 | | 0.11 | 0.01 | 0.23 | 0.01 | 2.89 | 0 | 0.07 | 0.15 | 0 | 0.01 |
| Stereum annosum | Stereaceae | 4360 | 0.4 | 0 | 2.41 | 0 | 38.26 | 0 | 52 | 7.04 | 0 | | 0.3 | 0 | 0.11 | 0 | 1.23 | 0 | 0.45 | 0.78 | 0 | 0.03 |

Supplementary Table S6. Bacterial genera and fungal species characterizing the different sites or groups of sites identified using hierarchical cluster analyses based on both bacterial and fungal community structures. We indicated potential associated environmental sources based on articles showing the presence (and mainly isolation) of the bacterial genus/fungal species in these potential sources.

| Site or group of sites | Bacterial genera | Potential source | References | Site or group of sites | Fungal species | Potential source | References |
| --- | --- | --- | --- | --- | --- | --- | --- |
| AMS | *Aquimarina* | Sea water | ^8,9^ | AMS | *Lophodermium agathidis* | Plants | ^10^ |
|  | *Ktedonobacter* | Soil | ^11^ |  | *Microdochium phragmitis* | Aquatic, plants | ^12^ |
|  | *Cocleimonas* | Sea water, sand snail | ^13,14^ |  | *Chalara hyalocuspica* | Plants | ^15^ |
|  | *Rubritalea* | Sea water, marine chordates | ^16–18^ |  | *Sistotremastrum guttuliferum* | Oceanic islands | ^19^ |
| STN | *Methylobacterium* | Soil, plants | ^20,21^ | STN | *Botryotinia fuckeliana (Botrytis cinerea)* | Plants | ^22^ |
|  | *Sediminibacterium* | Different environments (soil, sediment, reservoir) | ^23,24^ |  | *Bullera variabilis* | Plants | ^25^ |
|  | *Phenylobacterium* | Soil, rhizosphere, sludge | ^26,27^ |  | *Cryptococcus sp AL_V* |  |  |
|  | *Bradyrhizobium* | Plants | ^28^ |  | *Penicillium corylophilum* | Different environments (buildings) | ^29^ |
| CAP | *Sphingobacterium* | Soil, compost | ^30,31^ | CAP | *Cladosporium langeronii* | Human skin, insects | ^32^ |
|  | *Coraliomargarita* | Sea water | ^16^ |  | *Sporidiobolus sp JPS_2007a* |  |  |
|  | *Clostridium XI* | Feces-associated | ^33^ |  | *Entyloma dahlia* | Plants |  |
|  | *Streptomyces* | Soil | ^34,35^ |  | *Cladosporium salinae* | Saline environments | ^36^ |
| GRE/PDD | *Sphingomonas* | Different environments | ^37,38^ | GRE/PDD/PDM | *Alternaria sp BMV_2012* | Plants | ^39^ |
|  |  |  |  |  | *Coriolopsis troggi (trametes trogii)* | Soil |  |
| CHC/PDM/STP | *Segetibacter* | Soil | ^40^ | CHC/STP/NAM | *Ustilago bullata* | Plants | ^41^ |
| NAM | *Clostridium sensu-stricto* | Different environments |  |  | *Ustilago hordei* | Plants | ^42^ |
|  | *Modestobacter* | Surfaces, extreme conditions | ^43–45^ |  | *Toxicocladosporium rubrigenum* | Plants | ^46^ |
|  | *Blastococcus* | Different environments (sandstone, sea, soil pland, snow) | ^47^ |  |  |  |  |
|  | *Geminicoccus* | Biofilter of a marine aquaculture system | ^48^ |  |  |  |  |

Supplementary Table S7. Average concentration (and standard deviation) of the chemicals per site in ng/m^3^ of air. Part of these data was published in *Dommergue et al., 2019*^49^.

| Site | Organic carbon | Elemental carbon | MSA | Cl | NO3 | SO4 | Oxalate | Na | NH4 | K | Mg | Ca | Inositol | Glycerol | Erythriol | Xylitol | Mannitol + arabitol | Sorbitol | Threalose | Levoglucosan + mannosan | Galactosan | Rhamnose | Glucose |
| --- | --- | --- | --- | --- | --- | --- | --- | --- | --- | --- | --- | --- | --- | --- | --- | --- | --- | --- | --- | --- | --- | --- | --- |
| AMS | 1.96 ± 0.09 | 0 | 1.2 ± 0.19 | 3.99 ± 0.16 | 1.66 ± 0.16 | 3.13 ± 0.16 | 1.05 ± 0.11 | 3.73 ± 0.15 | 0.14 ± 0.27 | 2.27 ± 0.17 | 2.66 ± 0.16 | 2.35 ± 0.2 | < 0.007 | 0.08 ± 0.17 | 0.02 ± 0.02 | 0 ± 0.01 | 0.21 ± 0.16 | < 0.007 | 0.1 ± 0.08 | < 0.01 | < 0.007 | < 0.02 | 0.34 ± 0.22 |
| CAP | 2.4 ± 0.14 | 0.01 ± 0.01 | 1.69 ± 0.11 | 3.71 ± 0.17 | 2.8 ± 0.2 | 3.2 ± 0.11 | 1.72 ± 0.2 | 3.53 ± 0.11 | 2.05 ± 0.23 | 2.11 ± 0.08 | 2.46 ± 0.11 | 2.26 ± 0.24 | < 0.008 | 0.56 ± 0.16 | 0.16 ± 0.11 | 0.11 ± 0.09 | 0.22 ± 0.16 | 0.28 ± 0.12 | < 0.02 | 0.24 ± 0.23 | 0.01 ± 0.01 | < 0.02 | 0.21 ± 0.15 |
| CHC | 3.04 ± 0.14 | 0.04 ± 0.01 | 0.86 ± 0.09 | 1.06 ± 0.39 | 2.1 ± 0.6 | 2.99 ± 0.31 | 1.85 ± 0.16 | 1.47 ± 0.45 | 2.52 ± 0.24 | 1.77 ± 0.16 | 0.85 ± 0.25 | 1.79 ± 0.21 | 0.14 ± 0.1 | 0.57 ± 0.36 | 0.33 ± 0.11 | < 0.07 | 0.55 ± 0.09 | < 0.06 | < 0.035 | 1.63 ± 0.3 | 0.62 ± 0.32 | 0.01 ± 0.02 | 0.6 ± 0.14 |
| GRE | 3.55 ± 0.09 | 0.22 ± 0.05 | 1.54 ± 0.16 | 1.03 ± 0.36 | 2.51 ± 0.22 | 3.15 ± 0.14 | 2.29 ± 0.19 | 2.16 ± 0.27 | 2.16 ± 0.29 | 2.02 ± 0.13 | 1.42 ± 0.19 | 2.45 ± 0.22 | 0.24 ± 0.07 | < 0.30 | 0.51 ± 0.12 | 0.13 ± 0.08 | 1.58 ± 0.22 | 0.57 ± 0.14 | 1.07 ± 0.22 | 0.97 ± 0.24 | 0.02 ± 0.03 | < 0.02 | 1.3 ± 0.23 |
| NAM | 2.9 ± 0.15 | 0.04 ± 0.01 | 0.55 ± 0.11 | 0.87 ± 0.39 | 2.33 ± 0.45 | 2.81 ± 0.29 | 2.03 ± 0.13 | 1.81 ± 0.34 | 1.89 ± 0.3 | 1.28 ± 0.23 | 1.19 ± 0.26 | 2.31 ± 0.41 | 0.09 ± 0.06 | < 0.96 | 0.13 ± 0.05 | < 0.05 | 0.51 ± 0.23 | 0.04 ± 0.11 | 0.61 ± 0.15 | 0.69 ± 0.09 | 0.06 ± 0.02 | < 0.05 | 1.03 ± 0.3 |
| PDD | 3.12 ± 0.11 | 0.05 ± 0.01 | 1.4 ± 0.31 | 1.26 ± 0.63 | 2.61 ± 0.34 | 2.98 ± 0.11 | 2.16 ± 0.12 | 2.23 ± 0.21 | 2.42 ± 0.25 | 1.62 ± 0.13 | 1.42 ± 0.18 | 1.97 ± 0.25 | 0.2 ± 0.16 | < 0.16 | 0.39 ± 0.13 | 0.39 ± 0.16 | 1.22 ± 0.21 | 0.88 ± 0.19 | 0.78 ± 0.28 | 0.62 ± 0.21 | 0.05 ± 0.07 | < 0.01 | 0.98 ± 0.2 |
| PDM | 3.06 ± 0.21 | 0.03 ± 0.02 | 0.93 ± 0.34 | 1.05 ± 0.32 | 2.74 ± 0.3 | 2.92 ± 0.23 | 2.15 ± 0.22 | 1.97 ± 0.2 | 2.3 ± 0.34 | 1.48 ± 0.27 | 1.37 ± 0.23 | 2.38 ± 0.3 | 0.19 ± 0.11 | 0.11 ± 0.28 | 0.36 ± 0.18 | 0.18 ± 0.14 | 0.72 ± 0.24 | 0.93 ± 0.25 | 0.72 ± 0.25 | 0.4 ± 0.24 | 0.03 ± 0.05 | < 0.04 | 0.64 ± 0.25 |
| STN | 2.15 ± 0.23 | 0.01 ± 0 | 0.94 ± 0.23 | 1.28 ± 0.74 | 1.51 ± 0.39 | 2.67 ± 0.35 | 0.92 ± 0.42 | 1.72 ± 0.5 | 2.01 ± 0.24 | 0.79 ± 0.32 | 0.94 ± 0.42 | 1.38 ± 0.38 | 0.02 ± 0.01 | 0.1 ± 0.14 | 0 ± 0.01 | 0.01 ± 0.02 | 0.02 ± 0.04 | < 0.006 | < 0.008 | 0.02 ± 0.05 | < 0.006 | < 0.02 | 0.04 ± 0.06 |
| STP | 3.08 ± 0.5 | 0.02 ± 0.01 | 0.85 ± 0.33 | 0.58 ± 0.34 | 2.12 ± 0.58 | 2.3 ± 1.03 | 2.02 ± 0.48 | 1.22 ± 0.53 | 1.98 ± 0.58 | 1.36 ± 0.57 | 1.06 ± 0.45 | 1.83 ± 0.59 | 0.16 ± 0.1 | 0.33 ± 0.27 | 0.59 ± 0.3 | 0.12 ± 0.1 | 1.3 ± 0.6 | < 0.05 | 1.31 ± 0.6 | 0.74 ± 0.58 | 0.16 ± 0.18 | < 0.05 | 0.97 ± 0.44 |

Supplementary Table S8. Multiple linear regression results.

| Response variable | Explanatory variables | *R* squared | Adjusted *R* squared | Pvalue (*P*) |
| --- | --- | --- | --- | --- |
| Airborne bacterial community temporal variability  (similarity index) | -Wind direction variability within weeks  -Wind direction variability between weeks  -Landscape evenness (Pielou’s evenness)  -Temperature variability between weeks | 0.93 | 0.82 | 0.06 |
| Airborne fungal community temporal variability  (similarity index) | -Maximum wind speed  -Temperature variability between weeks | 0.87 | 0.83 | 0.002 |

**References**

1. Fierer, N., Jackson, J. A., Vilgalys, R. & Jackson, R. B. Assessment of Soil Microbial Community Structure by Use of Taxon-Specific Quantitative PCR Assays. *Appl. Environ. Microbiol.* **71**, 4117–4120 (2005).

2. Chemidlin Prévost-Bouré, N. *et al.* Validation and application of a PCR primer set to quantify fungal communities in the soil environment by real-time quantitative PCR. *PLoS ONE* **6**, e24166 (2011).

3. Masella, A. P., Bartram, A. K., Truszkowski, J. M., Brown, D. G. & Neufeld, J. D. PANDAseq: paired-end assembler for illumina sequences. *BMC Bioinformatics* **13**, 31 (2012).

4. Wang, Q., Garrity, G. M., Tiedje, J. M. & Cole, J. R. Naive Bayesian Classifier for Rapid Assignment of rRNA Sequences into the New Bacterial Taxonomy. *Applied and Environmental Microbiology* **73**, 5261–5267 (2007).

5. Burrows, S. M. *et al.* Bacteria in the global atmosphere – Part 2: Modeling of emissions and transport between different ecosystems. *Atmospheric Chemistry and Physics* **9**, 9281–9297 (2009).

6. Friedl, M. A. *et al.* Global land cover mapping from MODIS: algorithms and early results. *Remote Sensing of Environment* **83**, 287–302 (2002).

7. Burrows, S. M., Elbert, W., Lawrence, M. G. & Pöschl, U. Bacteria in the global atmosphere – Part 1: Review and synthesis of literature data for different ecosystems. *Atmos. Chem. Phys.* **9**, 9263–9280 (2009).

8. Nedashkovskaya, O. I. *et al.* Description of Aquimarina muelleri gen. nov., sp. nov., and proposal of the reclassification of [Cytophaga] latercula Lewin 1969 as Stanierella latercula gen. nov., comb. nov. *Int. J. Syst. Evol. Microbiol.* **55**, 225–229 (2005).

9. Yi, H. & Chun, J. Aquimarina addita sp. nov., isolated from seawater. *INTERNATIONAL JOURNAL OF SYSTEMATIC AND EVOLUTIONARY MICROBIOLOGY* **61**, 2445–2449 (2011).

10. Ortiz-García, S. *et al.* Phylogenetics of Lophodermium from Pine. *Mycologia* **95**, 846–59 (2003).

11. Wang, H. *et al.* Distribution and diversity of bacterial communities and sulphate-reducing bacteria in a paddy soil irrigated with acid mine drainage. *J. Appl. Microbiol.* **121**, 196–206 (2016).

12. Liu, Y., Zachow, C., Raaijmakers, J. M. & De Bruijn, I. Elucidating the Diversity of Aquatic Microdochium and Trichoderma Species and Their Activity against the Fish Pathogen Saprolegnia diclina. *International Journal of Molecular Sciences* **17**, 140 (2016).

13. Tanaka, N., Romanenko, L. A., Iino, T., Frolova, G. M. & Mikhailov, V. V. Cocleimonas flava gen. nov., sp. nov., a gammaproteobacterium isolated from sand snail (Umbonium costatum). *INTERNATIONAL JOURNAL OF SYSTEMATIC AND EVOLUTIONARY MICROBIOLOGY* **61**, 412–416 (2011).

14. Zeng, Y., Yu, Y., Qiao, Z.-Y., Jin, H.-Y. & Liu, Q. Diversity of bacterioplankton in coastal seawaters of Fildes Peninsula, King George Island, Antarctica. *Archives of microbiology* **196**, (2014).

15. Koukol, O. New species of Chalara occupying coniferous needles. *Fungal Diversity* **49**, 75 (2011).

16. Yoon, J. *et al.* Cerasicoccus arenae gen. nov., sp. nov., a carotenoid-producing marine representative of the family Puniceicoccaceae within the phylum ‘Verrucomicrobia’, isolated from marine sand. *Int. J. Syst. Evol. Microbiol.* **57**, 2067–2072 (2007).

17. Kasai, H. *et al.* Rubritalea squalenifaciens sp. nov., a squalene-producing marine bacterium belonging to subdivision 1 of the phylum ‘Verrucomicrobia’. *Int. J. Syst. Evol. Microbiol.* **57**, 1630–1634 (2007).

18. Scheuermayer, M. Rubritalea marina gen. nov., sp. nov., a marine representative of the phylum ‘Verrucomicrobia’, isolated from a sponge (Porifera). *INTERNATIONAL JOURNAL OF SYSTEMATIC AND EVOLUTIONARY MICROBIOLOGY* **56**, 2119–2124 (2006).

19. Telleria, M. *et al.* Sistotremastrum guttuliferum: A new species from the Macaronesian islands. *Mycological Progress* (2013). doi:10.1007/s11557-012-0876-0

20. Knief, C., Frances, L. & Vorholt, J. A. Competitiveness of diverse Methylobacterium strains in the phyllosphere of Arabidopsis thaliana and identification of representative models, including M. extorquens PA1. *Microb. Ecol.* **60**, 440–452 (2010).

21. Renier, A. *et al.* Nodulation of Crotalaria podocarpa DC. by Methylobacterium nodulans displays very unusual features. *J Exp Bot* **62**, 3693–3697 (2011).

22. Debieu, D., Bach, J., Hugon, M., Malosse, C. & Leroux, P. The hydroxyanilide fenhexamid, a new sterol biosynthesis inhibitor fungicide efficient against the plant pathogenic fungus Botryotinia fuckeliana (Botrytis cinerea). *Pest Management Science* **57**, 1060–1067 (2001).

23. Kang, H., Kim, H., Lee, B.-I., Joung, Y. & Joh, K. Sediminibacterium goheungense sp. nov., isolated from a freshwater reservoir. *Int. J. Syst. Evol. Microbiol.* **64**, 1328–1333 (2014).

24. Qu, J.-H. & Yuan, H.-L. Sediminibacterium salmoneum gen. nov., sp. nov., a member of the phylum Bacteroidetes isolated from sediment of a eutrophic reservoir. *Int. J. Syst. Evol. Microbiol.* **58**, 2191–2194 (2008).

25. Nakase, T. *et al.* Bullera begoniae sp. nov. and Bullera setariae sp. nov., two new species of ballistoconidium-forming yeasts in the Bullera variabilis (Bulleribasidium) cluster isolated from plants in Taiwan. *Mycoscience* **45**, 287–294 (2004).

26. Farh, M. E.-A., Kim, Y.-J., Singh, P., Hoang, V.-A. & Yang, D.-C. Phenylobacterium panacis sp. nov., isolated from the rhizosphere of rusty mountain ginseng. *Int. J. Syst. Evol. Microbiol.* **66**, 2691–2696 (2016).

27. Kanso, S. & Patel, B. K. C. Phenylobacterium lituiforme sp. nov., a moderately thermophilic bacterium from a subsurface aquifer, and emended description of the genus Phenylobacterium. *Int. J. Syst. Evol. Microbiol.* **54**, 2141–2146 (2004).

28. Nguyen, H. D. T., Cloutier, S. & Bromfield, E. S. P. Complete Genome Sequence of Bradyrhizobium ottawaense OO99T, an Efficient Nitrogen-Fixing Symbiont of Soybean. *Microbiol Resour Announc* **7**, e01477-18 (2018).

29. McMullin, D. R., Nsiama, T. K. & Miller, J. D. Secondary metabolites from Penicillium corylophilum isolated from damp buildings. *Mycologia* **106**, 621–628 (2014).

30. Yoo, S.-H. *et al.* Sphingobacterium composti sp. nov., isolated from cotton-waste composts. *Int. J. Syst. Evol. Microbiol.* **57**, 1590–1593 (2007).

31. Wei, W., Zhou, Y., Wang, X., Huang, X. & Lai, R. Sphingobacterium anhuiense sp. nov., isolated from forest soil. *Int. J. Syst. Evol. Microbiol.* **58**, 2098–2101 (2008).

32. Sun, T. *et al.* A Lethal Fungus Infects the Chinese White Wax Scale Insect and Causes Dramatic Changes in the Host Microbiota. *Scientific Reports* **8**, (2018).

33. Kubasova, T. *et al.* Effects of host genetics and environmental conditions on fecal microbiota composition of pigs. *PLOS ONE* **13**, e0201901 (2018).

34. Bentley, S. D. *et al.* Complete genome sequence of the model actinomycete Streptomyces coelicolor A3(2). *Nature* **417**, 141–147 (2002).

35. Higginbotham, S. J. & Murphy, C. D. Identification and characterisation of a Streptomyces sp. isolate exhibiting activity against methicillin-resistant Staphylococcus aureus. *Microbiological Research* **165**, 82–86 (2010).

36. Zalar, P. *et al.* Phylogeny and ecology of the ubiquitous saprobe Cladosporium sphaerospermum, with descriptions of seven new species from hypersaline environments. *Stud Mycol* **58**, 157–183 (2007).

37. Kaur, J., Kaur, J., Niharika, N. & Lal, R. Sphingomonas laterariae sp. nov., isolated from a hexachlorocyclohexane-contaminated dump site. *INTERNATIONAL JOURNAL OF SYSTEMATIC AND EVOLUTIONARY MICROBIOLOGY* **62**, 2891–2896 (2012).

38. Koskinen, R. *et al.* Characterization of Sphingomonas isolates from Finnish and Swedish drinking water distribution systems. *Journal of Applied Microbiology* **89**, 687–696 (2000).

39. Eram, D., Arthikala, M.-K., Melappa, G. & Santoyo, G. Alternaria species: endophytic fungi as alternative sources of bioactive compounds. *Italian Journal of Mycology* **47**, 40–54 (2018).

40. An, D.-S., Lee, H.-G., Im, W.-T., Liu, Q.-M. & Lee, S.-T. Segetibacter koreensis gen. nov., sp. nov., a novel member of the phylum Bacteroidetes, isolated from the soil of a ginseng field in South Korea. *Int. J. Syst. Evol. Microbiol.* **57**, 1828–1833 (2007).

41. Meyer, S. E., Nelson, D. L., Clement, S. & Ramakrishnan, A. Ecological genetics of the Bromus tectorum (Poaceae)-Ustilago bullata (Ustilaginaceae) pathosystem: A role for frequency-dependent selection? *Am. J. Bot.* **97**, 1304–1312 (2010).

42. Oksanen, J. *et al.* Community Ecology Package. (2019).

43. Sghaier, H. *et al.* Stone-dwelling actinobacteria *Blastococcus saxobsidens*, *Modestobacter marinus* and *Geodermatophilus obscurus* proteogenomes. *The ISME Journal* **10**, 21–29 (2016).

44. Normand, P. *et al.* Genome sequence of radiation-resistant Modestobacter marinus strain BC501, a representative actinobacterium that thrives on calcareous stone surfaces. *J. Bacteriol.* **194**, 4773–4774 (2012).

45. Busarakam, K. *et al.* Modestobacter caceresii sp. nov., novel actinobacteria with an insight into their adaptive mechanisms for survival in extreme hyper-arid Atacama Desert soils. *Syst. Appl. Microbiol.* **39**, 243–251 (2016).

46. Bezerra, J. D. P. *et al.* New endophytic Toxicocladosporium species from cacti in Brazil, and description of Neocladosporium gen. nov. *IMA Fungus* **8**, 77–97 (2017).

47. Castro, J. F. *et al.* Blastococcus atacamensis sp. nov., a novel strain adapted to life in the Yungay core region of the Atacama Desert. *Int. J. Syst. Evol. Microbiol.* **68**, 2712–2721 (2018).

48. Foesel, B. U., Gössner, A. S., Drake, H. L. & Schramm, A. Geminicoccus roseus gen. nov., sp. nov., an aerobic phototrophic Alphaproteobacterium isolated from a marine aquaculture biofilter. *Syst. Appl. Microbiol.* **30**, 581–586 (2007).

49. Dommergue, A. *et al.* Methods to investigate the global atmospheric microbiome. *Front. Microbiol.* **10**, (2019).
